# Incidence and mortality rates of strokes in Kazakhstan in 2014–2019

**DOI:** 10.1038/s41598-022-20302-8

**Published:** 2022-09-26

**Authors:** Gulnur Zhakhina, Bakhytbek Zhalmagambetov, Arnur Gusmanov, Yesbolat Sakko, Sauran Yerdessov, Elzar Matmusaeva, Aliya Imanova, Byron Crape, Antonio Sarria-Santamera, Abduzhappar Gaipov

**Affiliations:** 1 Department of Medicine, Nazarbayev University School of Medicine, Kerey and Zhanibek street 5/1, 010000 Nur-Sultan City, Republic of Kazakhstan; 2 Department of Neurology, Multidisciplinary City Hospital #2, Nur-Sultan, Kazakhstan; 3 Clinical Academic Department of Internal Medicine, CF “University Medical Center”, Nur-Sultan, Kazakhstan

**Keywords:** Epidemiology, Risk factors

## Abstract

There is a lack of information on the epidemiology of acute ischemic stroke (AIS), intracerebral hemorrhage (ICH), and subarachnoid hemorrhage (SAH) in developing countries. This research presents incidence and mortality rates of stroke patients based on hospital admission and discharge status in one of the Central Asian countries by analysis of large-scale healthcare data. The registry data of 177,947 patients admitted to the hospital with the diagnosis of stroke between 2014 and 2019 were extracted from the National Electronic Health System of Kazakhstan. We provide descriptive statistics and analyze the association of socio-demographic and medical characteristics such as comorbidities and surgical treatments. Among all stroke patients, the incidence rate based on hospital admission of AIS was significantly higher compared to SAH and ICH patients. In 5 year follow-up period, AIS patients had a better outcome than SAH and ICH patients (64.7, 63.1 and 57.3% respectively). The hazard ratio (HR) after the trepanation and decompression surgery was 2.3 and 1.48 for AIS and SAH patients; however, it was protective for ICH (HR = 0.87). The investigation evaluated an increase in the all-cause mortality rates based on the discharge status of stroke patients, while the incidence rate decreased over time.

## Introduction

Stroke is a serious non-communicable disorder ranking as a top second cause of death and disability around the world^1^. The World Health Organization defines stroke as “rapidly developing clinical signs of focal (or global) disturbance of cerebral function, with symptoms lasting 24 h or longer or leading to death, with no apparent cause other than of vascular origin”^2^.

Global Burden of Disease Stroke statistics revealed a crude incidence rate to be 185.01 per 100,000 person-years^3^. Specifically, the worldwide prevalence accounted for 101.5 million people, among which an ischemic subtype peaked up to 77.2 million, intracerebral one reached 20.7 million and subarachnoid hemorrhage was 8.4 million in 2019. In the same year, globally there were 3.3 million deaths due to ischemic stroke, 2.9 million due to intracerebral hemorrhage, and 0.4 million because of subarachnoid hemorrhage^4^.

Looking at Global trends, the absolute incidence of ischemic stroke has risen by 37%, the increase was displayed by hemorrhagic stroke accounting for 47%, while the total number of deaths due to both subtypes of stroke was leveled up approximately by 20%, between 1990 and 2010 years^5^. Therefore, the economic and healthcare burden of stroke will probably remain high.

Kazakhstan is a multi-ethnic developing country located in Central Asia with a population of about 19 million people. The majority of the population consists of ethnic Kazakhs (67.5%), Russians (19.5%), and others represented by Koreans, Uzbeks, Uyghurs, Ukrainians, and minority groups^6^. According to Akhmetzhanova et al.^7^, there were 58 specialized stroke centers accounted for by 2019 in Kazakhstan.

There is no large-scale whole population epidemiological data on stroke in Eurasian countries. Little is known about the epidemiology of stroke in Kazakhstan; however, Zhusupova et al.^8^, provided an estimate of the morbidity rate of cerebral stroke and accounted for 3.7 per 1000 person-years, and acute stroke was responsible for 52% of total morbidity. As for mortality rate, it was 1.08 per 1000 person-years and covered 26% of all mortality^8^.

Taking into account the current and possible future economic and healthcare burden of stroke, and the fact that epidemiologic parameters of the Kazakhstani population were never properly assessed, there is a clear need for accurate estimates. Therefore, the main objective of this study is to estimate the incidence and mortality rates based on hospital admission and discharge status of predominant stroke subtypes by using large-scale administrative data provided by the Unified National Electronic Health System (UNEHS) during the years 2014–2019.

## Methods

### Study design and population

This is a retrospective study, data extracted from the UNEHS between 2014 and 2019. The following the International Classification of Diseases (ICD) codes were considered a stroke events: acute ischemic stroke (I63 and I64), intracerebral hemorrhage (I61, I62.0, I62.1, and I62.9), and subarachnoid hemorrhage (I60)^9^. The raw data of 245,028 admissions between 2014 and 2019 were extracted from I60 to I69 codes related to cerebrovascular diseases. After data management and cleaning, 177,947 unique population registry numbers (RPN ID) were kept for further analysis (Supplementary Fig. 1). The number of population growth in Kazakhstan and its regions were obtained from the Statistics Committee under the Ministry of National Economy of the Republic of Kazakhstan^6^.

### Exposures and covariates

Individual patient data included date of birth, gender, ethnicity, address, ICD-10 diagnosis, date(s) and type(s) of admission, social status, type of surgical intervention, and death date if they were applicable. The birth and death dates were derived from the Population Registry through RPN ID. The address information was used to divide the living area into urban and rural. There were more than 100 nationalities; therefore, ethnicity was divided into 3: Kazakhs, Russians, and Others. The type of admission depended on the first admission to the hospital, either elective or urgent.

### Medical characteristics

Surgical interventions were categorized as endovascular, trepanation and decompression, neuronavigational operations, shunt and anastomosis, cardiovascular, and others. Definitions of surgery treatment options are given in Supplementary Table 1. The decision to perform surgical interventions should be made individually in each case as a result of discussion with the participation of neurologists, anesthesiologists, resuscitators, neurosurgeons, and vascular surgeons according to Kazakhstani clinical protocol for the disease treatment^10–12^.

The databases for diabetes and essential hypertension were formed based on information from the UNEHS. The criteria to define recurrence of stroke were adopted from the previously reported studies as a 21-day gap between the discharge day and the next admission day with any ICD-10 codes of stroke^13–15^. The death that occurred during the observation period was categorized as all-cause mortality, while the death during the hospitalization period was accounted as in-hospital mortality.

### Outcome assessment

Incidence and all-cause mortality rates based on hospital admission and discharge status were calculated by dividing new cases of stroke and death, respectively, by the total general population size by the end of each year. The death that occurred during the initial admission was accounted for in-hospital mortality. For analysis of all-cause mortality, the start date was defined as the day of the first admission, and the follow-up was until December 31st, 2019, or until the date of death.

### Statistical analysis

Incidence and all-cause mortality rates based on admission and discharge status were presented as per million population (PMP) in each administrative division of Kazakhstan. The maps were constructed for 2019, using QGIS 3.16.11 Hannover version. Data cleaning, data management, and statistical analysis were performed using STATA 16.1 MP2 version (STATA Corporation, College Station, TX).

The age-period cohort (APC) analysis was used to define the effect of age, period, and cohort on stroke incidence and mortality. The contribution of age is related to the changes in biological and social aspects due to aging. The period effect represents external factors during a specific period that equally affect all age categories. The cohort effect considers the unique exposure of a group as they move across time. APC framework allows to overcome the linear dependency (cohort = period-age) problem and evaluates their effect on trends and deviations. The APC analysis was done using R version 4.2.1.

Kaplan–Meier estimation and Log-rank tests were used to illustrate crude survival and statistical significance in survival by the type of diagnosis (AIS, ICH, and SAH). After checking the corresponding assumptions, Cox regression analyses were performed to demonstrate crude and adjusted hazard ratios. We constructed three sets of multivariable analysis models to test the adjusted effect of variables on mortality. The models were incrementally adjusted for potential confounders depending on theoretical background and their availability in the database. In the first model, only socio-demographic predictors (age, gender, ethnicity, living area, and admission type) were included. In the second model, we added diabetes and hypertension to Model 1. In the third model, surgical interventions were added to Model 2. In all models, the stepwise selection method was used. The fit of the models was evaluated by Akaike Information Criterion, Bayesian Information Criterion, and global goodness-of-fit test. The significance level was set at 0.05.

The study involved secondary data that was derived from the UNEHS, patients were not involved in the study. Therefore, the requirement for informed consent from study participants was waived by the Nazarbayev University Institutional Review Ethics Committee (NU-IREC 490/18112021). All methods were carried out in accordance with the “Reporting of studies conducted using observational routinely-collected health data” (RECORD) guideline.

### Ethics approval and consent to participate

The study was approved by the Nazarbayev University Institutional Review Ethics Committee (NU-IREC 490/18112021), with exemption from informed consent.

## Results

### Socio-demographic and baseline characteristics

The socio-demographic characteristics of the cohort (n = 177,947) are given in Table 1. During 2014–2019, there were 138,542 (78%) patients with acute ischemic stroke, 34,262 (19%) with intracerebral hemorrhage, and 5,143 (3%) diagnosed with subarachnoid hemorrhage. The cohort of ischemic stroke patients was the oldest (mean = 65 years). There were a total of 84,255 (47%) females and 93,692 (53%) males in the cohort. More than half (54%) of patients were ethnic Kazakhs, 28% were of Russian ethnicity and 18% were listed as other ethnicities. Overall, 169,962 (96%) patients of the whole cohort were admitted urgently to the hospital.

**Table 1:** Socio-demographic and medical characteristics of patients with different types of stroke for the years 2014–2019.

	Total (n = 177 947)	Acute ischemic stroke (n = 138 542, 78%)	Intracerebral hemorrhage (n = 34 262, 19%)	Subarachnoid hemorrhage (n = 5 143, 3%)
Age, (Mean ± SD)	63 ± 14.0	64.8 ± 12.7	59.2 ± 15.6	52 ± 19.7
**Age category, n (%)**				
< 18 y.o	1 727 (1)	426 (0.3)	898 (3)	403 (8)
18–34 y.o	2 947 (2)	1 809 (1.3)	809 (2)	329 (6)
35–50 y.o	20 858 (12)	14 040 (10)	5 645 (17)	1 173 (23)
51–70 y.o	97 296 (54)	75 434 (54.4)	19 350 (56)	2 512 (49)
> 70 y.o	55 119 (31)	46 833 (34)	7 560 (22)	726 (14)
**Gender, n (%)**				
Female	84 255 (47)	65 958 (48)	15 659 (46)	2 638 (51)
Male	93 692 (53)	72 584 (52)	18 603 (54)	2 505 (49)
**Ethnicity, n (%)**				
Kazakh	95 198 (54)	70 217 (51)	21 668 (63)	3 313 (64)
Russian	50 189 (28)	41 978 (30)	7 191 (21)	1 020 (20)
Other	32 560 (18)	26 347 (19)	5 403 (16)	810 (16)
**Living area, n (%)**				
Urban	112 644 (63)	90 106 (65)	19 779 (58)	2 759 (54)
Rural	65 303 (37)	48 436 (35)	14 483 (42)	2 384 (46)
**Admission, n (%)**				
Elective	7 985 (4)	6 309 (5)	1 351 (4)	325 (6)
Urgent	169 962 (96)	132 233 (95)	32 911 (96)	4 818 (94)
**Social status, n (%)**				
Employed	29 729 (17)	21 442 (15)	6 939 (20)	1 348 (26)
Unemployed	29 191 (16)	20 675 (15)	7 300 (22)	1 216 (24)
Retiree	101 141 (57)	83 682 (60)	15 718 (46)	1 741 (34)
Disabled	6 286 (4)	5 011 (4)	1 165 (3)	110 (2)
Other	11 600 (6)	7 732 (6)	3 140 (9)	728 (14)
**All-cause mortality, n (%)**				
Alive	117 887 (66)	94 133 (68)	20 444 (60)	3 310 (64)
In-hospital mortality, n (%)	23 368 (13)	13 368 (9.6)	8 789 (25.6)	1 211 (23.5)
**Surgery treatment, n (%)**				
Endovascular	4 615 (3)	3 319 (2.4)	810 (2.4)	486 (9.5)
Trepanation and decompression	2 421 (1.3)	98 (0.07)	2 259 (6.6)	64 (1.2)
Neuronavigational operation	310 (0.2)	16 (0.01)	286 (0.8)	8 (0.2)
Shunt and anastomosis	188 (0.1)	27 (0.02)	147 (0.4)	14 (0.3)
Cardiovascular	943 (0.5)	528 (0.4)	385 (1.1)	30 (0.6)
Other	1 897 (1.1)	1 745 (1.3)	127 (0.4)	25 (0.5)
No surgery	167 573 (93.8)	132 809 (95.8)	30 248 (88.3)	4 516 (87.7)
Recurrence, n (%)	10 012 (6)	7 204 (5)	2 465 (7)	343 (7)
Diabetes, n (%)	23 418 (13)	20 956 (15)	2 217 (6)	245 (5)
Hypertension, n (%)	86 218 (48)	70 477 (51)	13 919 (21)	1 822 (35)

Only 10,374 (6.2%) of the overall cohort received surgical treatment. Substantially there were endovascular operations (4,615 (3%)) and trepanation and decompression (2,421 (1.3%)). There were 10,012 (6%) recurrences in the overall cohort. 23 418 (13%) of the cohort had diabetes as a concurring disease, and 86,218 (48%) had essential hypertension. Over 2014–2019 years there were 60,060 (34%) deaths in the overall cohort. Of them, 23 368 (39%) account for in-hospital mortality cases.

### Incidence and mortality rates PMP based on admission and discharge status

**Figure 1:**
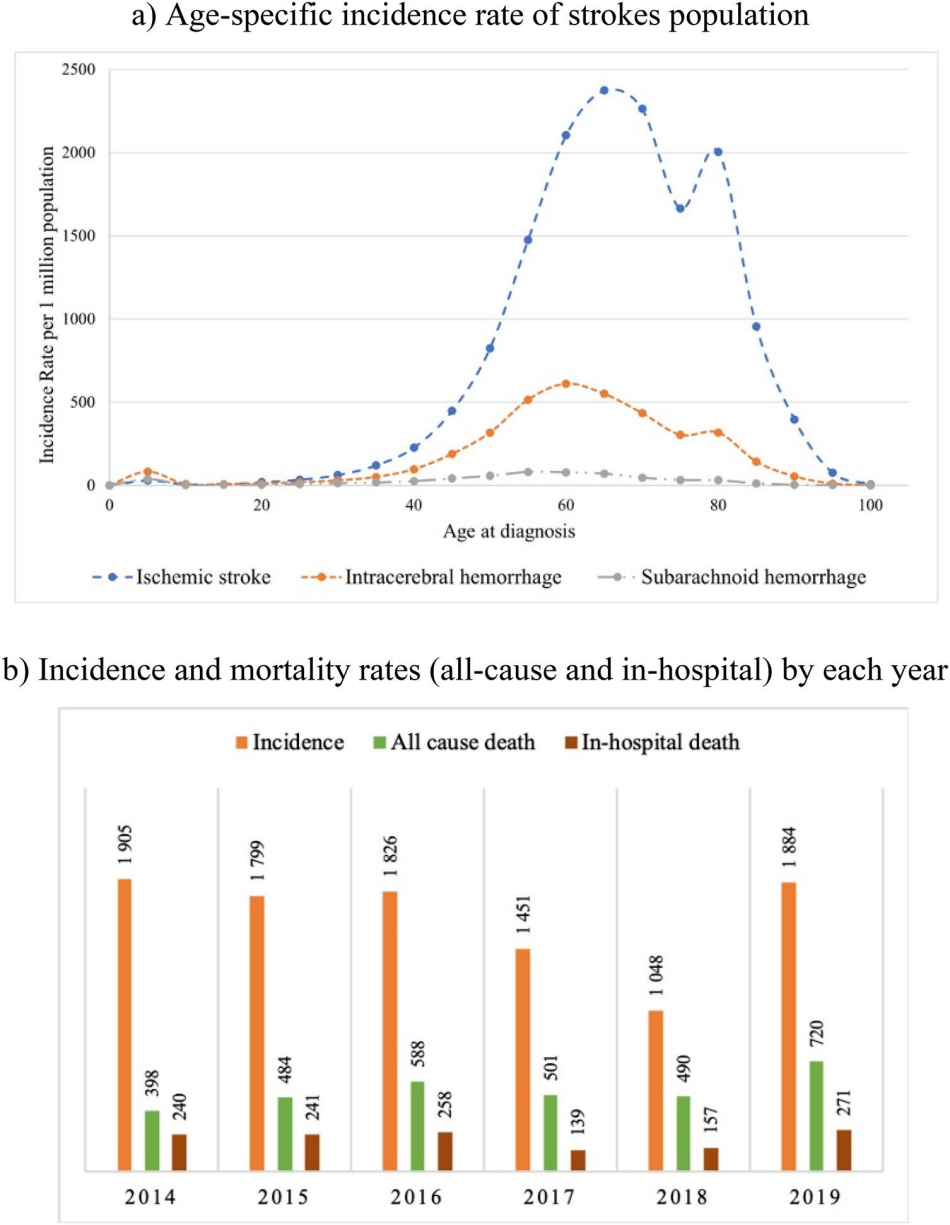
Incidence and mortality rates per 1,000,000 among stroke patients in Kazakhstan for the years 2014–2019: (**a**) Age-specific incidence rate of strokes population (**b**) Incidence and mortality rates (all-cause and in-hospital) by each year.

**Figure 2:**
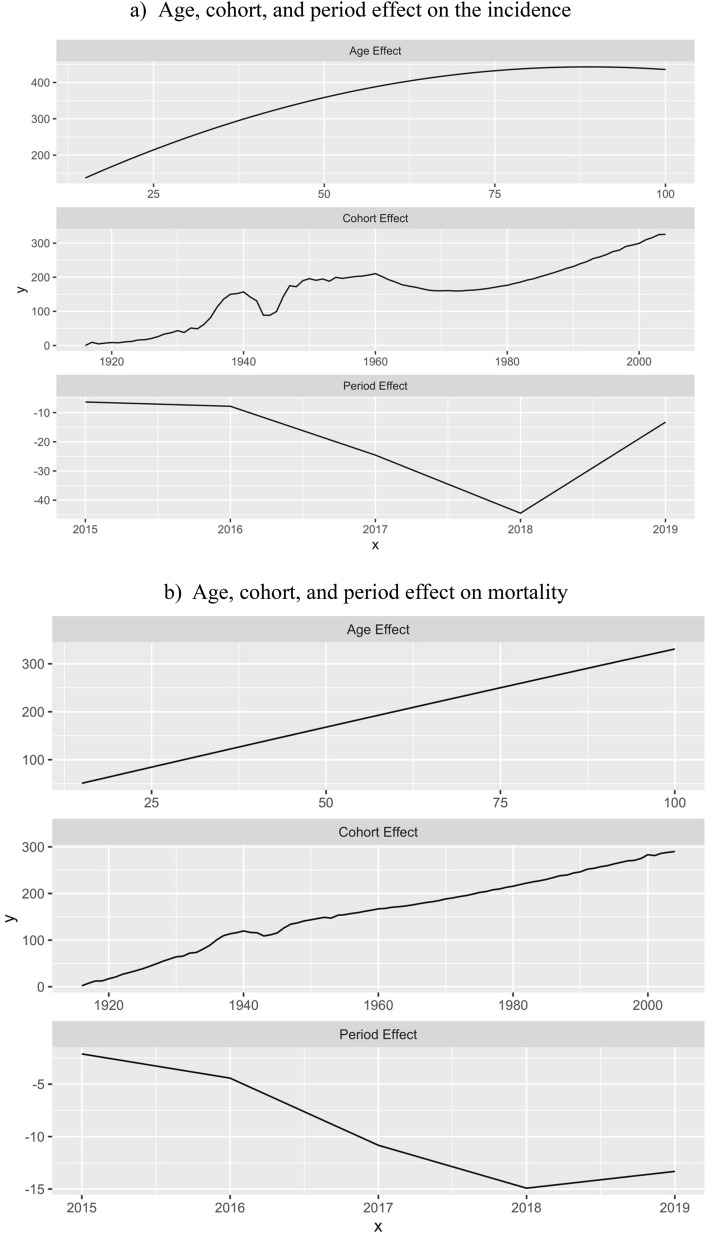
Age-period-cohort analysis: (**a**) Age, cohort, and period effect on incidence (**b**) Age, cohort, and period effect on mortality.

**Figure 3:**
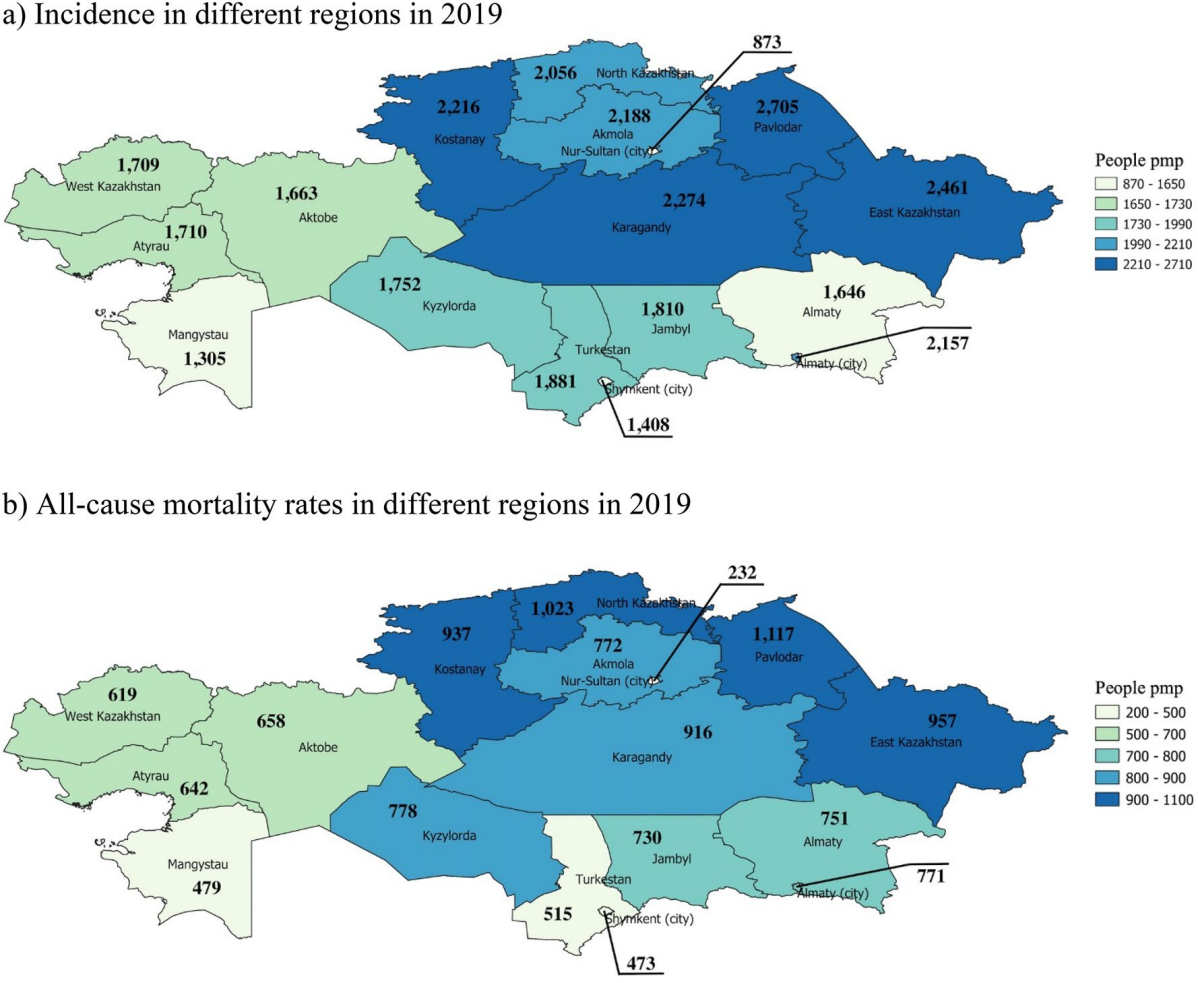
Incidence and all-cause mortality of stroke patients in Kazakhstan based on admission and discharge in 2019 (maps were generated using QGIS 3.16.11 Hannover version. URL: https://www.qgis.org): (**a**) Incidence in different regions in 2019 (**b**) All-cause mortality rates in different regions in 2019.

The highest age-specific incidence rate (IR) for AIS is at 65 years with 2,373 people per million population (PMP) (Fig. 1a), while for ICH and SAH are at 60 years (610 PMP) and 55 years (79 PMP) respectively. All-cause mortality rate based on discharge status increased from 398 to 720 PMP over the observation period. The in-hospital mortality rate increased from 240 PMP in 2014 to 271 PMP in 2019 (Fig. 1b). However, in 2017 and 2018 the rate sharply decreased to 139 PMP and 157 PMP respectively. The APC model shows an almost linear correlation between morbidity and mortality and aging (Fig. 2). Moreover, the period effect shows a sharp decrease in mortality of stroke patients from 2016 to 2018 (Fig. 2b). Incidence and all-cause mortality by the end of 2019 were higher predominantly in the Central, North, and East Kazakhstan regions (Fig. 3). The detailed information about incidence and all-cause mortality based on admission and discharge status by each type of stroke is given in Supplementary Figs. 2–4.

### Survival of stroke patients

**Figure 4:**
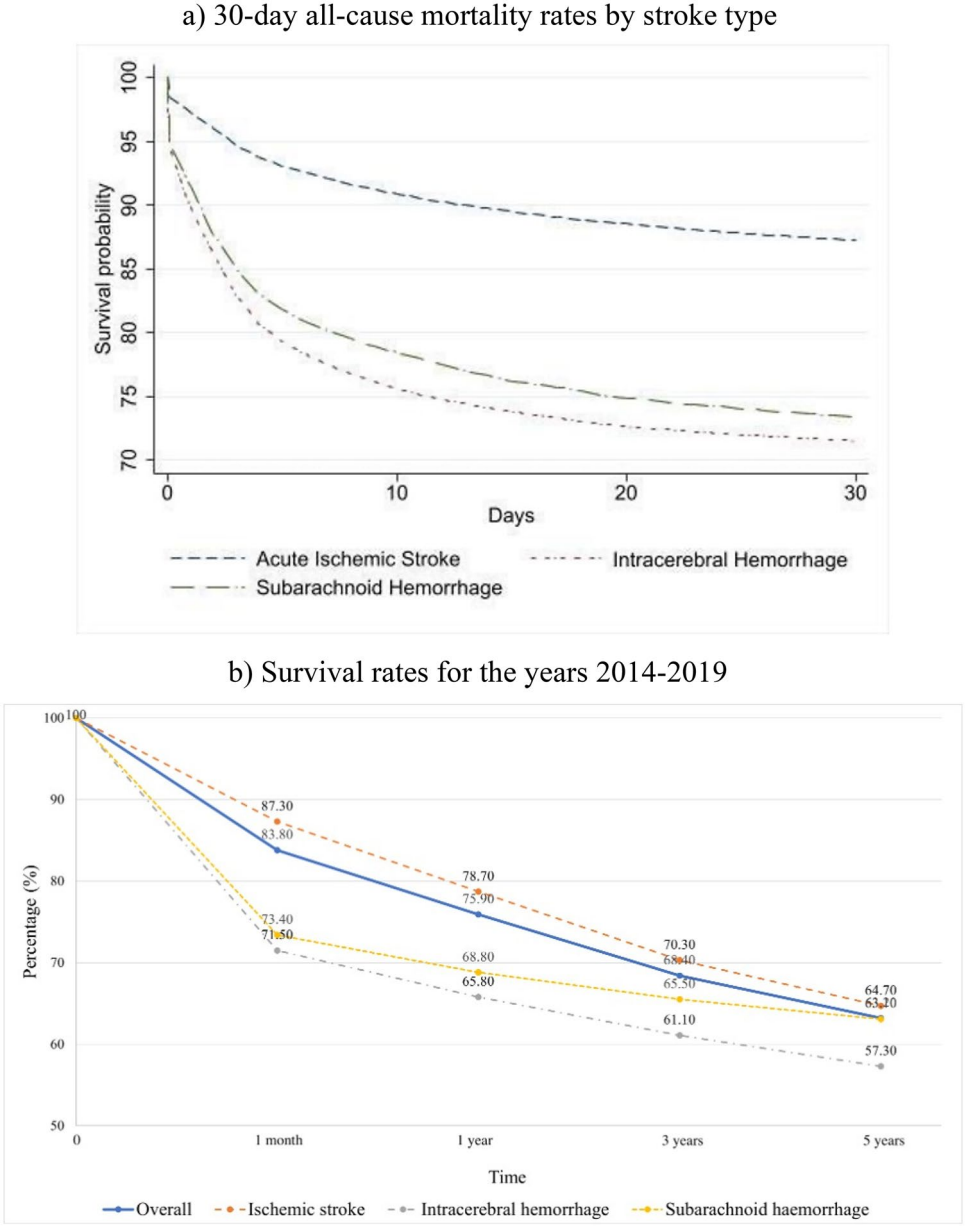
Kaplan–Meier survival curves due to all-cause mortality based on discharge status: (**a**) 30-day all-cause mortality rates by stroke type (**b**) Survival rates for the years 2014–2019.

Crude 30-day survival (Fig. 4a) of SAH and ICH patients is approximately the same (73.4 and 71.5% respectively). However, after adjustment for age and gender, the survival of SH patients is 95.3%, while for ICH patients is 91.9%. The survival tendency of 1-month in-hospital case-fatality is the same as for 1-month all-cause mortality (Supplementary Fig. 5). Although the 1-month crude survival rate for AIS patients is 87.3%, there is a sharp decrease to 64.7% by the 5-year follow-up period (Fig. 4b). From the perspective of a 5-year follow-up period, SAH patients have a better outcome than ICH patients (63.1 and 57.3% respectively).

### Hazard ratio by predictors

The Cox proportional hazard analysis showed that all variables except age younger than 35 years old were independent predictors of all-cause mortality in the cohort (Table 2). However, a more detailed analysis by stoke types showed that neuronavigational operations were not significant for AIS and SAH patients’ survival, the living area was not associated with hazard rates of ICH patients, and diabetes did not affect remarkably the mortality of SAH patients (Table 3). A more comprehensive investigation of in-hospital, 30-day, and all-cause mortality by stroke types is presented in Supplementary Tables 2–4.

**Table 2:** Association between socio-demographic and medical parameters and all-cause mortality rates in the cohort for the years 2014–2019.

Variable	Frequency/dead	Unadjusted	p-value	Model 1	p-value	Model 2	p-value	Model 3	p-value
	N/ n (%)	HR (95% CI)		HR (95% CI)		HR (95% CI)		HR (95% CI)	
**Demographics**									
Age category (< 18 y.o. (ref))									
18–34 y.o	2 947/ 438 (15)	0.91 (0.79; 1.06)	0.239	0.86 (0.74; 0.99)	0.046	0.91 (0.79; 1.06)	0.238	0.96 (0.83; 1.12)	0.593
35–50 y.o	20 858/ 4 123 (20)	1.26 (1.11; 1.42)	< 0.001	1.17 (1.04; 1.33)	0,01	1.45 (1.28; 1.64)	< 0.001	1.52 (1.35; 1.72)	< 0.001
51–70 y.o	97 296/ 27 290 (28)	1.86 (1.65; 2.09)	< 0.001	1.67 (1.48; 1.88)	< 0.001	2.17 (1.92; 2.44)	< 0.001	2.27 (2.01; 2.55)	< 0.001
> 70 y.o	55 119/ 27 933 (51)	3.93 (3.49; 4.42)	< 0.001	3.39 (3.02; 3.83)	< 0.001	4.19 (3.72; 4.72)	< 0.001	4.39 (3.9; 4.96)	< 0.001
Gender (Male vs Female (ref))	93 692/ 30 692 (33)	0.92 (0.91; 0.94)	< 0.001	1.1 (1.08; 1.12)	< 0.001	1.1 (1.07; 1.1)	< 0.001	1.08 (1.06; 1.1)	< 0.001
Ethnicity (Kazakh (ref))									
Russian	50 189/ 21 655 (43)	1.7 (1.67; 1.73)	< 0.001	1.46 (1.44; 1.49)	< 0.001	1.45 (1.43; 1.48)	< 0.001	1.45 (1.42; 1.47)	< 0.001
Other	32 560/ 11 520 (35)	1.31 (1.28; 1.34)	< 0.001	1.16 (1.14; 1.19)	< 0.001	1.12 (1.09; 1.15)	< 0.001	1.11 (1.09; 1.14)	< 0.001
Living area (Rural vs Urban (ref))	65 303/ 21 163 (32)	0.92 (0.91; 0.94)	< 0.001	1.08 (1.06; 1.09)	< 0.001	1.06 (1.05; 1.09)	< 0.001	1.07 (1.05; 1.09)	< 0.001
Admission (Urgent vs Elective (ref))	169 962/ 58 223 (34)	1.83 (1.75; 1.92)	< 0.001	1.55 (1.48; 1.62)	< 0.001	1.56 (1.48; 1.63)	< 0.001	1.55 (1.48; 1.63)	< 0.001
**Comorbidities**									
Diabetes	23 418/ 8 314 (36)	1.05 (1.03; 1.08)	< 0.001			1.25 (1.22; 1.28)	< 0.001	1.26 (1.23; 1.29)	< 0.001
Hypertension	86 218/ 22 586 (26)	0.57 (0.56; 0.58)	< 0.001			0.55 (0.54; 0.56)	< 0.001	0.56 (0.55; 0.57)	< 0.001
**Surgery types**									
Endovascular	4 615/ 881 (19)	0.53 (0.49; 0.57)	< 0.001					0.62 (0.58; 0.66)	< 0.001
Trepanation and decompression	2 421/ 926 (38)	1.23 (1.16; 1.32)	< 0.001					1.38 (1.29; 1.47)	< 0.001
Neuronavigational operation	310/ 74 (24)	0.68 (0.54; 0.86)	0.001					0.75 (0.59; 0.94)	0.013
Shunt and anastomosis	188/ 85 (45)	1.43 (1.15; 1.77)	0.001					1.81 (1.46; 2.24)	< 0.001
Cardiovascular	943/ 642 (68)	2.53 (2.34; 2.73)	< 0.001					2.26 (2.09; 2.44)	< 0.001

Model 1 = adjusted to demographics (age, gender, ethnicity, admission, residency); Model 2 = Model 1 + comorbidities; Model 3 = Model 2 + surgery types.

**Table 3:** Association between socio-demographic and medical parameters and all-cause mortality rates from stroke types for the years 2014–2019.

Variable	Acute Ischemic Stroke			Intracerebral Hemorrhage			Subarachnoid Hemorrhage		
	Frequency/dead, N/n (%)	HR (95% CI)	*p* value	Frequency/dead, N/n (%)	HR (95% CI)	*p* value	Frequency/dead, N/n (%)	HR (95% CI)	*p* value
**Demographics**									
Age category (< 18 y.o. (ref))									
18–34 y.o	1,809/165 (9)	1.08 (0.75;1.54)	0.696	809/ 200 (25)	1.24 (1.02;1.52)	0.034	329/73 (22)	1.76 (1.24;2.49)	0.001
35–50 y.o	14,040/18,99 (14)	1.86 (1.34;2.59)	< 0.001	5,645/1,888 (33)	2.04 (1.75;2.37)	< 0.001	1 173/336 (29)	2.81 (2.11;3.73)	< 0.001
51–70 y.o	75,434/19,076 (25)	3.71 (2.67;5.14)	< 0.001	19,350/7,286 (38)	2.32 (1.99;2.69)	< 0.001	2 512/928 (37)	4.07 (3.1;5.35)	< 0.001
> 70 y.o	46,833/23,233 (50)	8.15 (5.87;11.3)	< 0.001	7,560/4,261 (56)	3.37 (2.9;3.92)	< 0.001	726/439 (60)	6.6 (4.98;8.75)	< 0.001
Gender (Male vs Female (ref))	72,584/22,431 (31)	1.12 (1.1;1.14)	< 0.001	18,603/7,352 (40)	1.03 (0.99;1.07)	0.052	2 505/909 (36)	1.1 (1.01;1.21)	0.038
Ethnicity (Kazakh (ref))									
Russian	41,978/17,481 (42)	1.53 (1.49;1.57)	< 0.001	7,191/3,728 (52)	1.42 (1.36;1.48)	< 0.001	1,020/446 (44)	1.29 (1.15;1.45)	< 0.001
Other	26,347/8,969 (34)	1.18 (1.15;1.21)	< 0.001	5,403/2,236 (41)	1.05 (0.99;1.1)	0.059	810/315 (39)	1.07 (0.94;1.21)	0.303
Living area (Rural vs Urban (ref))	48,436/14,587 (30)	1.05 (1.03;1.07)	< 0.001	14,483/5,688 (39)	1.02 (0.99;1.06)	0.191	2,384/888 (37)	1.11 (1.01;1.23)	0.025
Admission (Urgent vs Elective (ref))	132,233/42,894 (32)	1.35 (1.28;1.42)	< 0.001	32,911/13,549 (41)	2.36 (2.1;2.67)	< 0.001	4,818/1,780 (37)	2.48 (1.88;3.26)	< 0.001
**Comorbidities**									
Diabetes	20,956/7,284 (35)	1.34 (1.3;1.37)	< 0.001	2,217/948 (43)	1.26 (1.18;1.35)	< 0.001	245/82 (33)	1.02 (0.81;1.27)	0.897
Essential hypertension	70,477/17,893 (25)	0.58 (0.57;0.59)	< 0.001	13,919/4,237 (30)	0.55 (0.53;0.57)	< 0.001	1,822/456 (25)	0.47 (0.42;0.52)	< 0.001
**Surgery types**									
Endovascular	3,319/617 (19)	0.63 (0.58;0.69)	< 0.001	810/187 (23)	0.55 (0.48;0.64)	< 0.001	486/ 77 (16)	0.42 (0.33;0.53)	< 0.001
Trepanation and decompression	98/53 (54)	2.3 (1.75;3.01)	< 0.001	2,259/838 (37)	0.87 (0.81;0.94)	< 0.001	64/35 (55)	1.48 (1.05;2.07)	0.023
Neuronavigational operation	16/5 (31)	0.77 (0.32;1.85)	0.56	286/66 (23)	0.49 (0.39;0.64)	< 0.001	8/3 (38)	1.68 (0.54;5.24)	0.369
Shunt and anastomosis	27/16 (59)	1.82 (1.11;2.98)	0.017	147/62 (42)	1.16 (0.9;1.49)	0.244	14/7 (50)	1.55 (0.73;3.26)	0.252
Cardiovascular	538/400 (74)	3.17 (2.88;3.51)	< 0.001	385/222 (58)	1.19 (1.04;1.36)	0.01	30/20 (67)	1.5 (0.96;2.34)	0.073

The highest mortality rate over the observation period was among ischemic stroke patients older than 70 years (HR = 8.15 [95%CI: 5.87–11.3]), while for intracerebral and subarachnoid hemorrhage patients HR were 3.37 [95%CI: 2.9–3.92] and 6.6 [95%CI 4.98–8.75] respectively after adjustment for all demographic and medical factors (Table 3). Having diabetes as comorbidity increased the risk of death of AIS patients by 34% (HR = 1.34 [95%CI: 1.3–1.37]) and ICH by 26% (HR = 1.26 [95%CI: 1.18–1.35]). Primarily hypertension decreased the risk of death from AIS by 42%, from ICH by 45%, and SH by 53% (*p* < 0.05). The highest ratio of 30-day mortality after admission to the hospital was observed among intracerebral and subarachnoid hemorrhage patients, who were admitted urgently to the hospital, 6.99 [95%CI: 5.42; 9.01] and HR 7.54 [95%CI: 4.27; 13.3] respectively (Supplementary Tables 3b and 4b).

## Discussion

This is the first study in Kazakhstan, even in Central Asia to investigate the incidence and mortality rates of acute ischemic stroke, intracerebral and subarachnoid hemorrhage based on admission and discharge status using nationwide large-scale administrative health data. The incidence dropped in 2017–2018, which can be attributed to a significant decrease of current tobacco smokers from 22.4% in 2014 to 20.4% in 2019 shown by the report Global Adult Tobacco Smoking Executive Summary^16^. Gattellari et al. studied the dynamics of admission and mortality rates in the Australian population using Big Data over nine years (2005–2014). They reported a non-significant decrease in admission rates for ischemic stroke, a significant decline in intracerebral hemorrhage, and stable rates for subarachnoid one^17^. Our analysis showed that people aged older than 50, male gender, Kazakh ethnicity, and living in urban areas were predominant in the cohort. A greater proportion of males in the cohort can be explained by the higher prevalence of risk factors among men compared to women^18,19^. Moreover, the studies defined differences in health-care-seeking behavior between gender, with women using medical services more frequently^20,21^. In addition, diabetes as comorbidity increased the risk of death; however, interestingly, essential hypertension was found to be protective for people who experienced a stroke. Surgical interventions had different effects on all-cause mortality rates depending on the stroke type.

In this retrospective study, we found that the incidence of stroke in 2019 was highest in Pavlodar, Karagandy, Kostanay, and East Kazakhstan regions and Almaty city. Modifiable risk factors that can lead to stroke episodes are high blood pressure, diabetes, smoking, obesity, and excessive alcohol use^22^. On the other hand, heredity, genetics, older age, gender, and race also can predict stroke^22,23^. Older people have a higher risk of atrial fibrillation, dislipidemia, or coronary heart disease, which are associated with increased stroke mortality. The results of environmental studies show increased air pollution due to heavy metal exposure, coal mines, industrial enterprises, and power production in the abovementioned regions except for Kostanay^24^, which could explain the increase in the incidence of stroke in these regions. Studies have shown that air pollution and traffic noise can be important environmental risk factors for stroke in urban societies^25,26^. The main pathophysiology behind environmental risk factors in increasing overall stroke mortality rates is linked to prolonged oxidative stress resulting in vascular endothelial dysfunction^25^.

The first episode of stroke was mostly experienced at 60–65 years of age, which is highly correlated with all-cause mortality. According to the aging index indicator, which characterizes the number of people aged 65 and over per 100 children younger than 15, North Kazakhstan and Kostanay regions have the highest odds of elderly people^6^. This might explain the high incidence and all-cause mortality among stroke patients PMP in these regions, but we need to be aware of medical care quality and other healthcare-related factors, which could significantly alter statistics in the low-income and developing countries such as Kazakhstan.

The highest rates of incidence and mortality in eastern and northern regions of Kazakhstan due to different types of strokes can be explained by health disparities and inequality. After the implementation of State Programs for Reforming and Development of Healthcare “Salamatty Kazakhstan”^27^ and “Densaulyk”^28^, the entire population of the country has equal access to an expanded primary health care system (PHC). The programs are dedicated to the prevention, early diagnosis, and treatment of all diseases including stroke. Moreover, the country practices a compulsory social health insurance system that allows maintaining the guaranteed volume of medical care that is provided free of charge^29^. Although the financing of the health care sector has increased, several problems remain unsolved that affect the unequal distribution of high-quality services. The lack of specialists, underdevelopment of long-term care and rehabilitation, poor infrastructure, and inadequate equipment in rural areas lead to health disparities^29^. Moreover, there is a study showing that the majority of rural settlements in north-eastern Kazakhstan have decreased access to PHC services^30^. In addition, Baikonur Cosmodrome and Semipalatinsk nuclear test sites are located in this region. These environmentally hazardous places have dramatically increased rates of cardiovascular diseases and cancer in the population living near them^31^.

In this research, we found that the crude one-month survival rate was the highest after AIS, followed by SAH and ICH. Current observation goes in line with stroke survival findings in the Lithuanian population^32^. Survival is lower in SAH compared to AIS due to the fact that complications after the former more seriously affect survival^33,34^. The worst survival rate was observed after ICH and becomes more evident even after adjusting for age and gender. One of the reasons why ICH has the highest mortality rates is due to the larger lesion size in ICH patients than in AIS. More specifically it was shown in the Copenhagen Stroke Study that the maximum diameter of lesions in individuals with ICH was 20% larger than the affected area in patients with AIS^35^.

Literature data on the effect of essential hypertension history on survival after stroke is various. For example, Hagos Gufue et al. did not find a statistically significant difference in survival among hypertensive and non-hypertensive patients following stroke^36^. Sami Tetri et al., showed that the history of primary hypertension alone was not responsible for early death following spontaneous intracerebral hemorrhage^37^. In contrast, Henriksson et al. found that hypertension was associated with decreased survival following hemorrhagic stroke as compared to ischemic one^38^. In general, survival outcomes are worse in hypertensive patients after stroke. To our best knowledge, there is no study showing the protective effects of essential hypertension on survival following stroke. In the current study, the history of primary hypertension was shown to be protective from death among all 3 types of stroke after adjustment for all demographic and medical characteristics. However, a more focused study on the association of essential hypertension with mortality in stroke patients should be analyzed in the hypertension cohort involving more clinical and laboratory data.

According to current results, diabetes as comorbidity significantly increases the risk of death in acute ischemic and intracerebral hemorrhage stroke patients. The fact that the mortality rate was higher after AIS than after ICH is not surprising. Salah-Eddine Megherbi et al., found that diabetic patients were statistically significantly more prone to be affected by cerebral infarction than hemorrhage^39^. Olsson et al. found that survival is lower in diabetic patients compared to non-diabetic^40^. Simpson et al. showed that diabetic status did not significantly alter the outcome after subarachnoid hemorrhage^41^. The current study also illustrated no significant difference in terms of survival after SAH in diabetic patients.

The results of this study show that endovascular surgeries were protective for all three types of stroke. Similar observations were found for SAH^42^, AIS^43^. Some ICH are caused by aneurysm rupture or vessel anomaly^44^, thus endovascular surgery techniques (clipping/coiling) are beneficial. Trepanation and decompression surgeries were protective for ICH and not protective for both AIS and SAH. In general, it is a common practice to treat traumatic brain injury, SAH, and middle cerebral artery (MCA) infarction with decompression. Nonetheless, there is no clear evidence about the effectiveness of decompression techniques as a treatment as the review article shows^45^. Turning to cardiovascular surgeries the mortality rates increased after AIS and ICH. Clearly, these surgeries were done on patients with cardiovascular pathology, and the risk of mortality is higher in patients with underlying cardiovascular impairments than in patients without. In addition, as Ko S.B. reports, perioperative stroke occurrence is higher during cardiac surgeries in comparison to non-cardiac surgeries^46^. Current data finds that shunting and anastomosis surgeries increased the risks of mortality after AIS. Reynolds et al. report that 21% of strokes were likely to be attributed to extracranial-intracranial (EC-IC) vascular bypass surgery^47^, of which were included in the shunt and anastomosis category. Although some of these findings might enrich the knowledge of the effect of different surgeries on survival, more studies are needed to investigate the long-term outcomes following post-stroke surgeries.

There are several limitations in this study such as the lack of information on comorbidities, such as obesity, atrial fibrillation, chronic kidney disease, family history of stroke, and smoking status, alcohol consumption which might affect the survival. In addition, the database does not have information on medications and treatment strategies. There could also be possible errors with disease coding, and a lack of data on the cause-specific mortality. In addition, the lack of details about rehabilitation measures (type, duration, frequency), also could affect survival. The effect of surgery types on survival was assessed as a combination into 5 major groups, based on their clinical relatedness, and thus there is a certain degree of internal variability of surgery types, which affects survival rates. Since calculations were based on admission and discharge numbers from the registry, for those who had a stroke but were not admitted to the hospital or died before admission, there could be an underestimation of the incidence and mortality rates.

Strengths derive from the fact that the source of data is a population-based registry, which provides sufficiently large, representative information, and a data collection period is adequately long to detect numerous stroke cases. Moreover, this is the first study from Central Asia to demonstrate the epidemiology of stroke, more specifically AIS, ICH, and SAH, which adds valuable information to the world literature. Besides the demographic factors, the effectiveness of surgical treatment for survival was evaluated. The findings will be valuable for further research in this field.

## Conclusion

This is the first study in Kazakhstan that presents the incidence and mortality rate of stroke events based on admission and discharge status. The current study retrospectively analyzed large-scale administrative healthcare data of stroke patients over five years between 2014 and 2019. The results showed that the incidence rate per year did not increase over the observed period, and the all-cause mortality rate among stroke patients approximately doubled in the observed period. Although the 1-month survival rate after the first episode of stroke was highest among acute ischemic stroke patients, in a perspective of 5 years, it was the same as for SAH patients. The lowest 5-year survival probability is observed for ICH patients. The paper also describes the hazard ratio of socio-demographic and medical characteristics of patients such as comorbidities and performed surgical treatment.

## Supplementary Information

Supplementary Information.

## Data Availability

The data that support the findings of this study are available from the Republican Center for Electronic Health of the Ministry of Health of the Republic of Kazakhstan but restrictions apply to the availability of these data, which were used under license for the current study, and so are not publicly available. Data are however available from the corresponding author, Gaipov A., upon reasonable request and with permission of the Ministry of Health of the Republic of Kazakhstan.

## Abbreviations

AIS: Acute ischemic stroke
HR: Hazard ratio
ICH: Intracerebral hemorrhage
IR: Incidence rate
PMP: Per million population
RPN: Population registry number
SAH: Subarachnoid hemorrhage
UNEHS: Unified National Electronic Health System
WHO: World Health Organization
