# Supplementary material for: Incidence and mortality rates of strokes in Kazakhstan in 2014–2019
Source: Sci Rep. 2022 Sep 26;12:16041. doi: 10.1038/s41598-022-20302-8 (PMC9512804; doi:10.1038/s41598-022-20302-8)

##### Supplementary table 1. Types of surgeries which were classified into 5 different categories

| **Surgery code** | **Surgery Name** | **Category** |
| --- | --- | --- |
| 00.63 | Percutaneous insertion of carotid artery stent(s) | Endovascular |
| 00.64 | Percutaneous insertion of extracranial vascular stents | Endovascular |
| 00.65 | Percutaneous insertion of intracranial vascular stent(s) | Endovascular |
| 00.66 | Percutaneous transluminal coronary angioplasty [PTCA] | Other |
| 00.93 | Transplant from cadaver | Other |
| 01.02 | Ventriculopuncture through previously implanted catheter | Shunt and anastomosis |
| 01.09 | Incision and excision of skull. brain. and cerebral meninges; Other cranial | Shunt and anastomosis |
| 01.10 | Intracranial pressure monitoring | Trepanation and decompression |
| 01.16 | Intracranial oxygen monitoring | Trepanation and decompression |
| 01.18 | Other diagnostic procedures on brain and cerebral meninges | Trepanation and decompression |
| 01.19 | Other diagnostic procedures on skull | Trepanation and decompression |
| 01.20 | Cranial implantation or replacement of neurostimulator pulse generator | Trepanation and decompression |
| 01.23 | Craniotomy and craniectomy; Reopening of craniotomy site | Trepanation and decompression |
| 01.24 | Other craniotomy | Trepanation and decompression |
| 01.25 | Other craniectomy | Trepanation and decompression |
| 01.26 | Insertion of catheter(s) into cranial cavity or tissue | Trepanation and decompression |
| 01.28 | Placement of intracerebral catheter(s) via burr hole(s) | Trepanation and decompression |
| 01.31 | Incision of cerebral meninges | Trepanation and decompression |
| 01.39 | Other incision of brain | Trepanation and decompression |
| 01.51 | Excision of lesion or tissue of cerebral meninges | Trepanation and decompression |
| 01.59 | Other excision or destruction of lesion or tissue of brain | Trepanation and decompression |
| 01.591 | Excision of damaged brain tissue using intraoperative neuromonitoring | Trepanation and decompression |
| 02.01 | Opening of cranial suture | Other |
| 02.03 | Formation of cranial bone flap | Other |
| 02.05 | Insertion of skull plate | Other |
| 02.06 | Other cranial osteoplasty | Other |
| 02.20 | Ventricilostomy | Trepanation and decompression |
| 02.24 | Anastomosis of ventricle to cervical subarachnoid space | Trepanation and decompression |
| 02.31 | Ventricular shunt to structure in head and neck | Shunt and anastomosis |
| 02.32 | Ventricular shunt to circulatory system | Shunt and anastomosis |
| 02.34 | Ventricular shunt to abdominal cavity and organs | Shunt and anastomosis |
| 02.39 | Vent shunt extracran NEC | Shunt and anastomosis |
| 02.391 | Ventriculostomy of the bottom of the 3rd ventricle (endoscopic) | Trepanation and decompression |
| 02.42 | Replacement of ventricular shunt | Shunt and anastomosis |
| 02.43 | Removal of ventricular shunt | Other |
| 02.99 | Other operations on skull. brain. and cerebral meninges; Other | Shunt and anastomosis |
| 03.09 | Other exploration and decompression of spinal canal | Other |
| 03.31 | Spinal tap | Other |
| 03.39 | Other diagnostic procedures on spinal cord and spinal canal structures | Other |
| 03.52 | Repair of spinal myelomeningocele | Other |
| 03.799 | neurosurgical navigational operations | Neuronavigational |
| 03.7991 | Spine and spinal cord operations with the use of neuronavigation | Other |
| 03.99 | Other operations on spinal cord and spinal canal structures; Other | Other |
| 04.2 | Destruction of cranial and peripheral nerves | Other |
| 07.65 | Total excision of pituitary gland. transsphenoidal approach | Other |
| 13.71 | Insertion of intraocular lens prosthesis at time of cataract extraction. one-stage | Other |
| 13.73 | Cataract phacoemulsification with iol implantation | Other |
| 17.39 | Other laparoscopic partial excision of large intestine | Other |
| 21.7 | Reduction of nasal fracture | Other |
| 21.901 | Opening of a nasal boil | Other |
| 28.2 | Tonsillectomy without adenoidectomy | Other |
| 28.6 | Adenoidectomy without tonsillectomy | Other |
| 31.00 | Injection of larynx | Other |
| 31.2 | Permanent tracheostomy | Other |
| 31.21 | Mediastinal tracheostomy | Other |
| 31.29 | Other permanent tracheostomy | Other |
| 32.1 | Other excision of bronchus | Other |
| 32.20 | Thoracoscopic excision of lesion or tissue of lung | Other |
| 32.4 | Lobectomy of lung | Other |
| 33.22 | Fiber-optic bronchoscopy | Other |
| 34.02 | Exploratory thoracotomy | Other |
| 34.04 | Insertion of intercostal catheter for drainage | Other |
| 34.06 | Thoracoscopic drainage of pleural cavity | Other |
| 34.28 | Other diagnostic procedures on chest wall. pleura. and diaphragm | Other |
| 34.59 | Other excision of pleura | Other |
| 34.91 | Thoracentesis | Other |
| 34.99 | Other operations on thorax | Other |
| 35.14 | Open heart valvuloplasty of tricuspid valve without replacement | Cardiovascular |
| 35.33 | Annuloplasty | Cardiovascular |
| 35.95 | Revision of corrective procedure on heart | Cardiovascular |
| 35.99 | Other operations on valves of heart | Cardiovascular |
| 35.991 | Clipping of the mitral orifice | Cardiovascular |
| 36.06 | Insertion of non-drug-eluting coronary artery stent(s) | Cardiovascular |
| 36.07 | Insertion of drug-eluting coronary artery stent(s) | Cardiovascular |
| 36.11 | (Aorto)coronary bypass of one coronary artery | Cardiovascular |
| 36.12 | (Aorto)coronary bypass of two coronary arteries | Cardiovascular |
| 36.13 | (Aorto)coronary bypass of three coronary arteries | Cardiovascular |
| 36.14 | (Aorto)coronary bypass of four or more coronary arteries | Cardiovascular |
| 37.0 | Pericardiocentesis | Cardiovascular |
| 37.32 | Excision of aneurysm of heart | Cardiovascular |
| 37.78 | Insertion of temporary transvenous pacemaker system | Cardiovascular |
| 37.94 | Implantation or replacement of automatic cardioverter/defibrillator. total | Cardiovascular |
| 38.02 | Incision of vessel; other vessels of head and neck | Cardiovascular |
| 38.03 | Incision of vessel; upper limb vessels | Cardiovascular |
| 38.08 | Incision of vessel; lower limb arteries | Cardiovascular |
| 38.11 | Endarterectomy; intracranial vessels | Cardiovascular |
| 38.12 | Endarterectomy; other vessels of head and neck | Cardiovascular |
| 38.121 | Endarteriectomy (carotid artery ans its branches. jugular vein) | Cardiovascular |
| 38.18 | Endarterectomy; lower limb arteries | Cardiovascular |
| 38.29 | Other diagnostic procedures on blood vessels | Cardiovascular |
| 38.31 | Resection of vessel with anastomosis; intracranial vessels | Cardiovascular |
| 38.59 | Ligation and stripping of varicose veins; lower limb veins | Cardiovascular |
| 38.61 | Other excision of vessel; intracranial vessels | Cardiovascular |
| 38.63 | Other excision of vessel; upper limb vessels | Cardiovascular |
| 38.81 | Other surgical occlusion of vessels; intracranial vessels | Cardiovascular |
| 38.89 | Other surgical occlusion of vessels; lower limb veins | Cardiovascular |
| 39.2 | Other shunt or vascular bypass | Cardiovascular |
| 39.25 | Aorta-iliac-femoral bypass | Cardiovascular |
| 39.27 | Arteriovenostomy for renal dialysis | Cardiovascular |
| 39.28 | Extracranial-intracranial (EC-IC) vascular bypass | Shunt and anastomosis |
| 39.29 | Other (peripheral) vascular shunt or bypass | Shunt and anastomosis |
| 39.31 | Suture of artery | Shunt and anastomosis |
| 39.49 | Other revision of vascular procedure | Shunt and anastomosis |
| 39.51 | Clipping of aneurysm | Endovascular |
| 39.511 | Clipping of cerebral vascular aneurysms | Endovascular |
| 39.512 | Coagulation or suturing of cerebral vessels (aneurysms) | Endovascular |
| 39.531 | Repair of arteriovenous fistula | Endovascular |
| 39.561 | Repair of blood vessel with tissue patch graft | Endovascular |
| 39.57 | Repair of blood vessel with synthetic patch graft | Endovascular |
| 39.59 | Other repair of vessel | Other |
| 39.7 | Endovascular procedures on vessel(s) | Endovascular |
| 39.72 | Endovascular (total) embolization or occlusion of head and neck vessels | Endovascular |
| 39.74 | Endovascular removal of obstruction from head and neck vessel(s) | Endovascular |
| 39.741 | Endovascular stenting of head and neck vessels | Endovascular |
| 39.75 | Endovascular embolization or occlusion of vessel(s) of head or neck using bare coils | Endovascular |
| 39.76 | Endovascular embolization or occlusion of vessel(s) of head or neck using bioactive coils | Endovascular |
| 39.79 | Other endovascular procedures on other vessels | Endovascular |
| 39.792 | Endovascular vascular stenting | Endovascular |
| 39.793 | Endovascular balloon vessels angioplasty | Endovascular |
| 39.7945 | Endovascular arteries embolization | Endovascular |
| 39.795 | Endovascular selective catheterization of arteries for long-term infusion therapy | Endovascular |
| 39.98 | Control of hemorrhage. not otherwise specified | Other |
| 39.99 | Other operations on vessels | Other |
| 41.1 | Puncture of spleen | Other |
| 41.5 | Total splenectomy | Other |
| 42.91 | Ligation of esophageal varices | Other |
| 43.0 | Gastrectomy | Other |
| 43.42 | Local excision of other lesion or tissue of stomach | Other |
| 43.6 | Partial gastrectomy with anastomosis to duodenum | Other |
| 43.7 | Partial gastrectomy with anastomosis to jejunum | Other |
| 44.29 | Other pyloroplasty | Other |
| 44.41 | Suture of gastric ulcer site | Other |
| 44.42 | Suture of duodenal ulcer site | Other |
| 44.64 | Gastropexy | Other |
| 44.69 | Other repair of stomach | Other |
| 44.99 | Other operations on stomach | Other |
| 45.31 | Other local excision of lesion of duodenum | Other |
| 45.33 | Local excision of lesion or tissue of small intestine. except duodenum | Other |
| 45.62 | Other partial resection of small intestine | Other |
| 45.75 | Open and other left hemicolectomy | Other |
| 46.21 | Temporary ileostomy | Other |
| 46.73 | Suture of laceration of small intestine. except duodenum | Other |
| 46.991 | Elimination of intestinal obstruction | Other |
| 47 | Operations on appendix | Other |
| 47.1 | Incidental appendectomy | Other |
| 49.01 | Incision of perianal abscess | Other |
| 49.12 | Anal fistulectomy | Other |
| 49.46 | Excision of hemorrhoids | Other |
| 50.61 | Closure of laceration of liver | Other |
| 50.9 | Other operations on liver | Other |
| 51 | Operations on gallbladder and biliary tract | Other |
| 51.01 | Percutaneous aspiration of gallbladder | Other |
| 51.03 | Other cholecystostomy | Other |
| 51.2 | Cholecystectomy | Other |
| 51.22 | Other cholecystectomy | Other |
| 51.23 | Laparoscopic cholecystectomy | Other |
| 51.391 | Percutaneous transhepatic cholecystocholangiostomy | Other |
| 51.43 | Insertion of choledochohepatic tube for decompression | Other |
| 51.87 | Endoscopic insertion of stent (tube) into bile duct | Other |
| 52.211 | Drainage of the lesser omentum | Other |
| 52.402 | Pancreatic cystogastrostomy | Other |
| 53.02 | Other and open repair of indirect inguinal hernia | Other |
| 53.03 | Other and open repair of direct inguinal hernia with graft or prosthesis | Other |
| 53.4 | Repair of umbilical hernia | Other |
| 53.51 | Incisional hernia repair | Other |
| 53.59 | Repair of other hernia of anterior abdominal wall | Other |
| 53.61 | Other open incisional hernia repair with graft or prosthesis | Other |
| 54.1 | Laparotomy | Other |
| 54.11 | Exploratory laparotomy | Other |
| 54.12 | Reopening of recent laparotomy site | Other |
| 54.19 | Other laparotomy | Other |
| 54.21 | Laparoscopy | Other |
| 54.5 | Lysis of peritoneal adhesions | Other |
| 54.51 | Laparoscopic lysis of peritoneal adhesions | Other |
| 54.95 | Incision of peritoneum | Other |
| 55.03 | Percutaneous nephrostomy without fragmentation | Other |
| 55.91 | Decapsulation of kidney | Other |
| 56.1 | Transurethral removal of obstruction from ureter | Other |
| 56.31 | Ureteroscopy | Other |
| 57 | Operations on urinary bladder | Other |
| 57.1 | Cystotomy and cystostomy | Other |
| 57.11 | Percutaneous aspiration of bladder | Other |
| 57.18 | Other suprapubic cystostomy | Other |
| 57.19 | Other cystotomy | Other |
| 57.51 | Excision of urachus | Other |
| 57.9 | Other operations on bladder | Other |
| 60.2 | Transurethral prostatectomy | Other |
| 60.202 | Bipolar transurethral resection of benign prostatic hyperplasia | Other |
| 60.3 | Suprapubic prostatectomy | Other |
| 68.3 | Subtotal abdominal hysterectomy | Other |
| 68.41 | Laparoscopic total abdominal hysterectomy | Other |
| 68.411 | Total laparoscopic hysterectomy | Other |
| 69.01 | Dilation and curettage for termination of pregnancy | Other |
| 69.02 | Dilation and curettage following delivery or abortion | Other |
| 69.09 | Other dilation and curettage of uterus | Other |
| 69.51 | Aspiration curettage of uterus for termination of pregnancy | Other |
| 69.52 | Aspiration curettage following delivery or abortion | Other |
| 74.0 | Classical cesarean section | Other |
| 76.76 | Open reduction of mandibular fracture | Other |
| 76.99 | Other operations on facial bones and joints | Other |
| 77.67 | Local excision of lesion or tissue of bone; tibia and fibula | Other |
| 78.15 | Application of external fixator device; femur | Other |
| 79.06 | Closed reduction of fracture without internal fixation; tibia and fibula | Other |
| 79.111-1 | Closed reduction of fracture with internal fixation; humerus | Other |
| 79.151-1 | Closed reduction of fracture with internal fixation; femur | Other |
| 79.191-1 | Closed reduction of fracture with internal fixation; other specified bone | Other |
| 79.31 | Open reduction of fracture with internal fixation; humerus | Other |
| 79.311 | Open reposition of bone fragments of the humerus with internal fixation by blocking intramedullary osteosynthesis | Other |
| 79.35 | Open reduction of fracture with internal fixation; femur | Other |
| 79.36 | Open reduction of fracture with internal fixation; tibia and fibula | Other |
| 79.39 | Open reduction of fracture with internal fixation; other specified bone | Other |
| 79.55 | Open reduction of separated epiphysis; femur | Other |
| 80.203 | Arthroscopic synovectomy of the joint | Other |
| 80.98 | Other excision of joint; foot and toe | Other |
| 81.51 | Total hip replacement | Other |
| 81.52 | Partial hip replacement | Other |
| 81.65 | Percutaneous vertebroplasty | Other |
| 83.14 | Fasciotomy | Other |
| 84.05 | Amputation through forearm | Other |
| 84.07 | Amputation through humerus | Other |
| 84.10 | Lower limb amputation. not otherwise specified | Other |
| 84.11 | Amputation of toe | Other |
| 84.12 | Amputation through foot | Other |
| 84.15 | Other amputation below knee | Other |
| 84.17 | Amputation above knee | Other |
| 84.65 | Insertion of total spinal disc prosthesis. lumbosacral | Other |
| 84.99 | Other operations on musculoskeletal system | Other |
| 86.0 | Incision of skin and subcutaneous tissue | Other |
| 86.04 | Other incision with drainage of skin and subcutaneous tissue | Other |
| 86.09 | Other incision of skin and subcutaneous tissue | Other |
| 86.11 | Closed biopsy of skin and subcutaneous tissue | Other |
| 86.19 | Other diagnostic procedures on skin and subcutaneous tissue | Other |
| 86.22 | Excisional debridement of wound. infection. or burn | Other |
| 86.221 | Surgical and debridement | Other |
| 86.222 | Opening and drainage of soft tissue abscess | Other |
| 86.3 | Other local excision or destruction of lesion or tissue of skin and subcuta | Other |
| 86.4 | Radical excision of skin lesion | Other |
| 88.41 | Arteriography of cerebral arteries | Endovascular |
| 88.42 | Aortography | Endovascular |
| 88.50 | Angiocardiography. not otherwise specified | Endovascular |
| 88.55 | Coronary arteriography using a single catheter | Other |
| 88.56 | Coronary arteriography using two catheters | Other |
| 88.59 | Intraoperative coronary fluorescence vascular ang | Endovascular |
| 98.5109 | Ureterolithotripsy with extraction | Other |
| 99.10 | Injection or infusion of thrombolytic agent | Other |
| 99.101 | Intravascular thrombolysis of cerebral arteries and sinuses | Endovascular |

Supplementary figure 1. Flow chart diagram of cohort set-up.


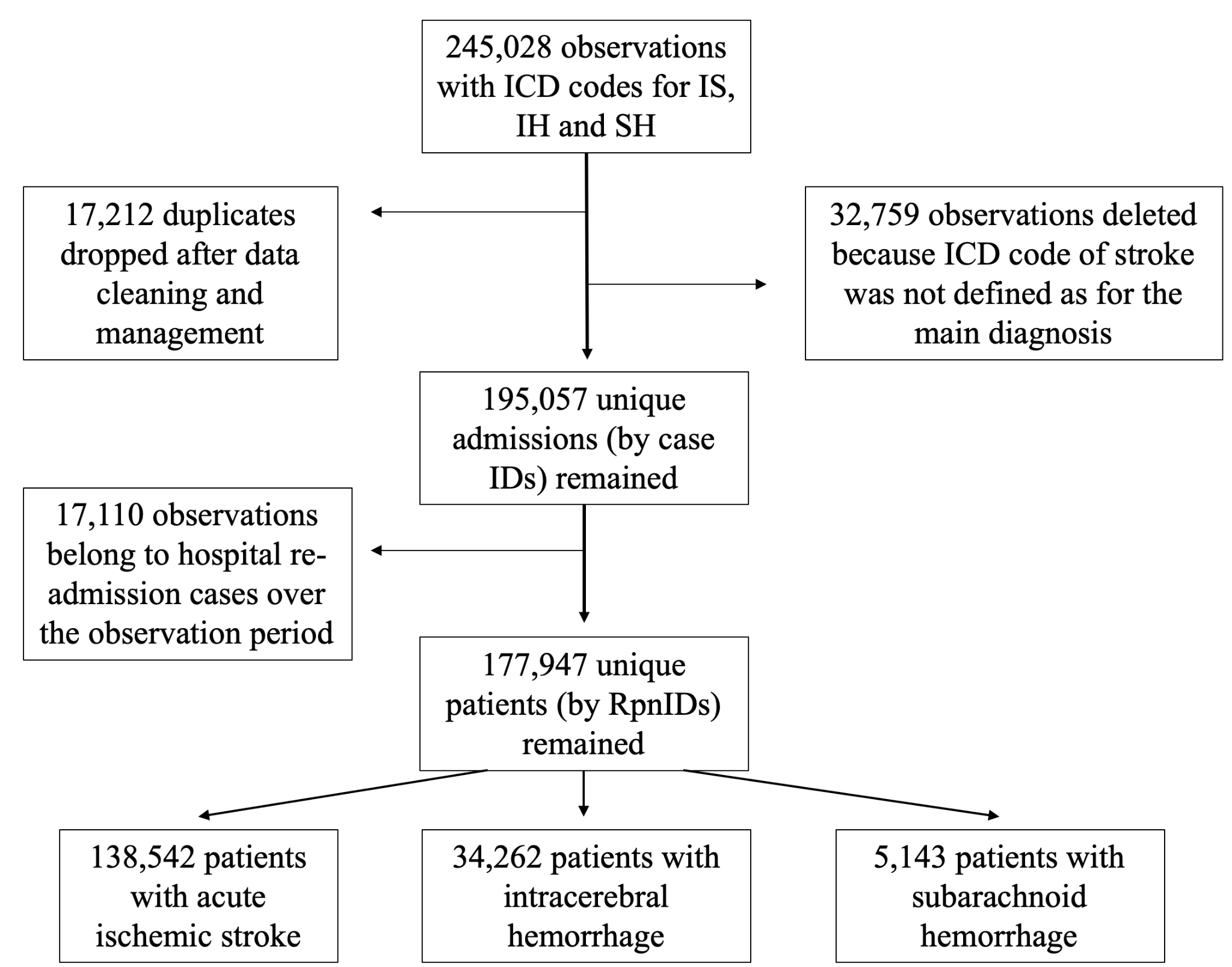


##### Supplementary figure 2. Acute ischemic stroke in Kazakhstan (maps were generated using QGIS 3.16.11 Hannover version. URL: https://www.qgis.org)

a) Incidence based on admission records by regions in 2019


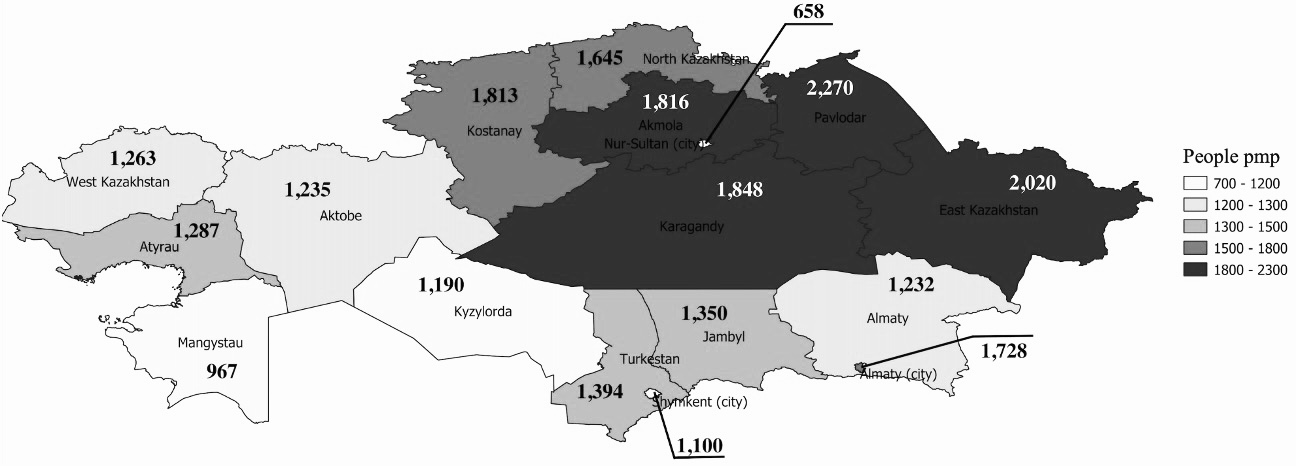


b) All-cause mortality rate of ischemic stroke patients based on discharge status in Kazakhstan in 2019


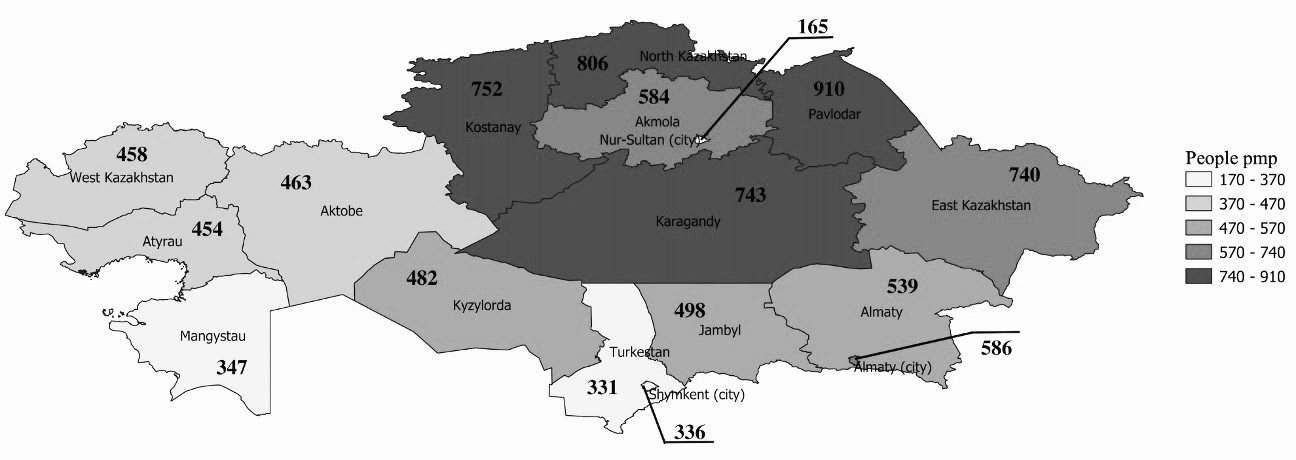


#####

##### Supplementary figure 3. Intracerebral hemorrhage in Kazakhstan (maps were generated using QGIS 3.16.11 Hannover version. URL: <https://www.qgis.org>)

a) Incidence based on admission records in different regions in 2019


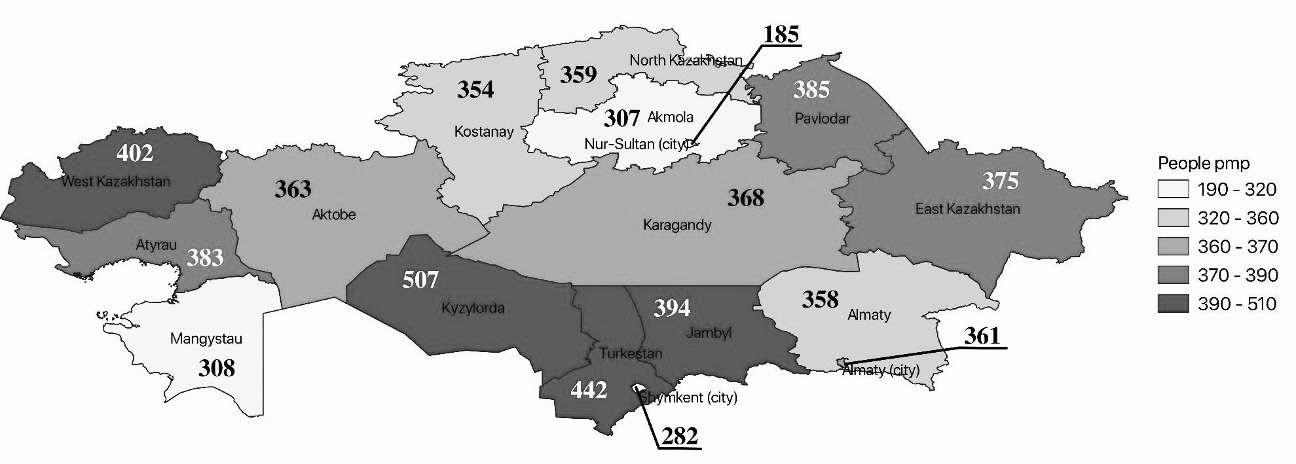


b) All-cause mortality rate of ischemic stroke patients based on discharge status in Kazakhstan in 2019


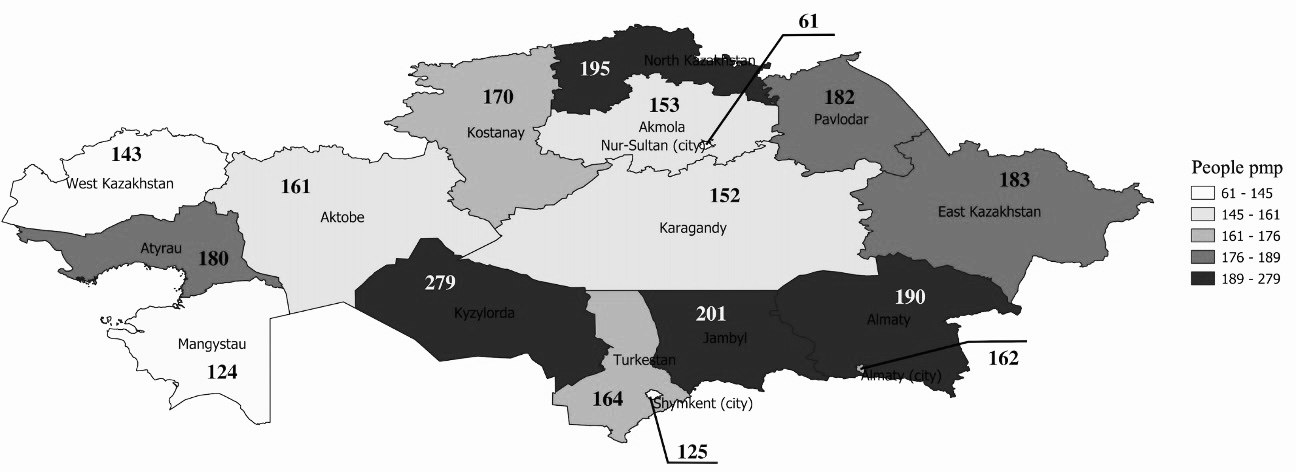


#####

##### Supplementary figure 4. Subarachnoid hemorrhage in Kazakhstan (maps were generated using QGIS 3.16.11 Hannover version. URL: https://www.qgis.org)

a) Incidence based on admission records in different regions in 2019


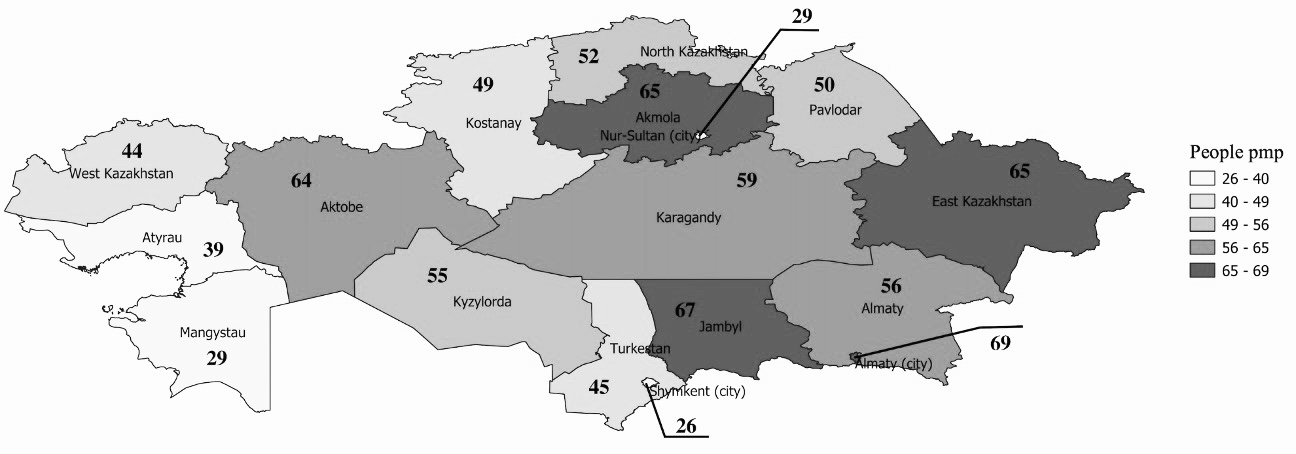


b) All-cause mortality rate of ischemic stroke patients based on discharge status in Kazakhstan in 2019


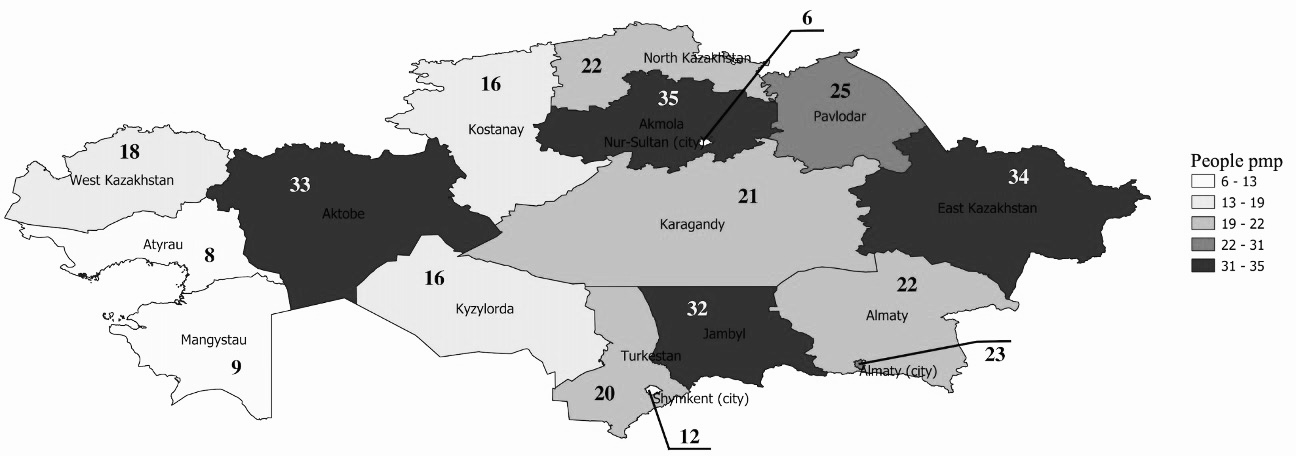


Supplementary table 2. Association between socio-demographics and medical parameters and mortality rates of ischemic stroke (n=138,542) in the years of 2014-2019

a) in-hospital case-fatality rates

| Variable | Frequency/dead | Unadjusted | p-value | Model 1 | p-value | Model 2 | p-value | Model 3 | p-value |
| --- | --- | --- | --- | --- | --- | --- | --- | --- | --- |
|  | N/ n (%) | HR (95% CI) |  | HR (95% CI) |  | HR (95% CI) |  | HR (95% CI) |  |
| **Demographics** | | | | | | | | |  |
| Age category (<18 y.o. (ref)) |  |  |  |  |  |  |  |  |  |
| 18 - 34 y.o. | 1 809/ 86 (4.8) | 0.77 (0.49; 1.19) | 0.245 | 0.72 (0.47; 1.12) | 0.146 | 0.79 (0.51; 1.23) | 0.293 | 0.80 (0.52; 1.25) | 0.329 |
| 35 - 50 y.o | 14 040/ 843 (6) | 0.98 (0.67; 1.45) | 0.927 | 0.92 (0.62; 1.35) | 0.661 | 1.25 (0.84; 1.84) | 0.27 | 1.26 (0.86; 1.87) | 0.238 |
| 51 - 70 y.o. | 75 434/ 6 018 (8) | 1.33 (0.91; 1.96) | 0.145 | 1.16 (0.79; 1.70) | 0.454 | 1.71 (1.16; 2.52) | 0.006 | 1.73 (1.18; 2.54) | 0.005 |
| > 70 y.o. | 46 833/ 6 673 (14.2) | 2.53 (1.72; 3.71) | <0.001 | 2.03 (1.38; 2.98) | <0.001 | 2.76 (1.88; 4.06) | <0.001 | 2.79 (1.89; 4.10) | <0.001 |
| Gender (Male vs Female (ref)) | 72 584/ 6 866 (9.5) | 0.91 (0.88; 0.94) | <0.001 | 1.09 (1.05; 1.13) | <0.001 | 1.06 (1.03; 1.10) | <0.001 | 1.06 (1.03; 1.09) | 0.001 |
| Ethnicity (Kazakh (ref)) |  |  |  |  |  |  |  |  |  |
| Russian | 41 978/ 5 813 (13.8) | 2.01 (1.93; 2.09) | <0.001 | 1.73 (1.66; 1.79) | <0.001 | 1.69 (1.62; 1.76) | <0.001 | 1.67 (1.61; 1.74) | <0.001 |
| Other | 26 347/ 2 762 (10.5) | 1.49 (1.42; 1.56) | <0.001 | 1.32 (1.26; 1.39) | <0.001 | 1.26 (1.20; 1.32) | <0.001 | 1.25 (1.19; 1.31) | <0.001 |
| Living area (Rural vs Urban (ref)) | 48 436/ 4 131 (8.5) | 0.79 (0.77; 0.83) | <0.001 | 0.97 (0.94; 1.01) | 0.162 | 0.95 (0.92; 0.99) | 0.014 | 0.95 (0.92; 0.99) | 0.014 |
| Admission (Urgent vs Elective (ref)) | 132 233/ 13 401 (10) | 2.77 (2.44; 3.14) | <0.001 | 2.27 (1.99; 2.57) | <0.001 | 2.29 (2.01; 2.59) | <0.001 | 2.29 (2.01; 2.59) | <0.001 |
| **Comorbidities** | | | | | | | | |  |
| Diabetes | 20 956/ 1 908 (9) | 0.91 (0.87; 0.96) | <0.001 |  |  | 1.18 (1.12; 1.24) | <0.001 | 1.18 (1.13; 1.25) | <0.001 |
| Hypertension | 70 477/ 4 225 (6) | 0.41 (0.39; 0.43) | <0.001 |  |  | 0.41 (0.39; 0.43) | <0.001 | 0.42 (0.40; 0.43) | <0.001 |
| **Surgery types** | | | | | | | | |  |
| Endovascular | 3 319/ 134 (4) | 0.39 (0.33; 0.46) | <0.001 |  |  |  |  | 0.44 (0.37; 0.52) | <0.001 |
| Trepanation and decompression | 98/ 44 (45) | 4.62 (3.44; 6.21) | <0.001 |  |  |  |  | 4.02 (2.98; 5.41) | <0.001 |
| Neuronavigational operation | 16/ 4 (25) | 1.87 (0.70; 4.99) | 0.21 |  |  |  |  | 1.32 (0.49; 3.54) | 0.578 |
| Shunt and anastomosis | 27/ 14 (52) | 5.21 (3.08; 8.79) | <0.001 |  |  |  |  | 3.94 (2.33; 6.67) | <0.001 |
| Cardiovascular | 538/ 266 (49.4) | 5.76 (5.10; 6.50) | <0.001 |  |  |  |  | 4.50 (3.98; 5.09) | <0.001 |

Model 1= adjusted to demographics (age, gender, ethnicity, admission, residency); Model 2 = Model 1 + comorbidities; Model 3 = Model 2 + surgery types.

b) 30-day all-cause mortality rates

| Variable | Frequency/dead | Unadjusted | p-value | Model 1 | p-value | Model 2 | p-value | Model 3 | p-value |
| --- | --- | --- | --- | --- | --- | --- | --- | --- | --- |
|  | N/ n (%) | HR (95% CI) |  | HR (95% CI) |  | HR (95% CI) |  | HR (95% CI) |  |
| **Demographics** | | | | | | | | |  |
| Age category (<18 y.o. (ref)) |  |  |  |  |  |  |  |  |  |
| 18 - 34 y.o. | 1 809/ 92 (5) | 1.03 (0.64; 1.65) | 0.91 | 0.96 (0.59; 1.55) | 0.874 | 1.06 (0.66; 1.69) | 0.816 | 1.08 (0.67; 1.73) | 0.759 |
| 35 - 50 y.o | 14 040/ 831 (6) | 1.20 (0.78; 1.86) | 0.402 | 1.13 (0.73; 1.74) | 0.583 | 1.57 (1.02; 2.42) | 0.041 | 1.59 (1.04; 2.46) | 0.034 |
| 51 - 70 y.o. | 75 434/ 6 763 (9) | 1.85 (1.20; 2.84) | 0.005 | 1.62 (1.05; 2.48) | 0.028 | 2.47 (1.61; 3.79) | <0.001 | 2.49 (1.63; 3.84) | <0.001 |
| > 70 y.o. | 46 833/ 9 912 (21.2) | 4.61 (3.00; 7.07) | <0.001 | 3.68 (2.39; 5.65) | <0.001 | 5.13 (3.34; 7.88) | <0.001 | 5.19 (3.38; 7.97) | <0.001 |
| Gender (Male vs Female (ref)) | 72 584/ 8 346 (11.5) | 0.81 (0.79; 0.83) | <0.001 | 1.03 (0.99; 1.06) | 0.091 | 0.99 (0.97; 1.03) | 0.874 | 0.99 (0.97; 1.03) | 0.819 |
| Ethnicity (Kazakh (ref)) |  |  |  |  |  |  |  |  |  |
| Russian | 41 978/ 7 632 (18.2) | 2.11 (2.04; 2.19) | <0.001 | 1.73 (1.67; 1.79) | <0.001 | 1.69 (1.63; 1.75) | <0.001 | 1.68 (1.62; 1.74) | <0.001 |
| Other | 26 347/ 3 673 (13.9) | 1.59 (1.53; 1.66) | <0.001 | 1.35 (1.29; 1.41) | <0.001 | 1.29 (1.24; 1.34) | <0.001 | 1.28 (1.22; 1.33) | <0.001 |
| Living area (Rural vs Urban (ref)) | 48 436/ 5 490 (11.3) | 0.83 (0.81; 0.86) | <0.001 | 1.05 (1.02; 1.09) | 0.004 | 1.02 (0.99; 1.06) | 0.212 | 1.02 (0.99; 1.05) | 0.235 |
| Admission (Urgent vs Elective (ref)) | 132 233/ 17 406 (13.2) | 4.09 (3.58; 4.69) | <0.001 | 3.19 (1.29; 3.65) | <0.001 | 3.22 (2.81; 3.69) | <0.001 | 3.23 (2.82; 3.69) | <0.001 |
| **Comorbidities** | | | | | | | | |  |
| Diabetes | 20 956/ 2 329 (11) | 0.85 (0.81; 0.89) | <0.001 |  |  | 1.12 (1.07; 1.17) | <0.001 | 1.12 (1.07; 1.18) | <0.001 |
| Hypertension | 70 477/ 5 119 (7.3) | 0.37 (0.36; 0.39) | <0.001 |  |  | 0.38 (0.37; 0.39) | <0.001 | 0.39 (0.37; 0.40) | <0.001 |
| **Surgery types** | | | | | | | | |  |
| Endovascular | 3 319/ 174 (5.2) | 0.39 (0.33; 0.45) | <0.001 |  |  |  |  | 0.46 (0.40; 0.54) | <0.001 |
| Trepanation and decompression | 98/ 47 (48) | 3.88 (2.91; 5.17) | <0.001 |  |  |  |  | 3.85 (2.89; 5.13) | <0.001 |
| Neuronavigational operation | 16/ 3 (18.8) | 1.09 (0.35; 3.39) | 0.886 |  |  |  |  | 1.02 (0.33; 3.16) | 0.977 |
| Shunt and anastomosis | 27/ 15 (55.6) | 4.42 (2.67; 7.34) | <0.001 |  |  |  |  | 3.50 (2.11; 5.82) | <0.001 |
| Cardiovascular | 538/ 285 (53) | 4.79 (4.26; 5.38) | <0.001 |  |  |  |  | 3.76 (3.34; 4.23) | <0.001 |

Model 1= adjusted to demographics (age, gender, ethnicity, admission, residency); Model 2 = Model 1 + comorbidities; Model 3 = Model 2 + surgery types.

c) all-cause mortality rates

| Variable | Total/dead | Unadjusted | p-value | Model 1 | p-value | Model 2 | p-value | Model 3 | p-value |
| --- | --- | --- | --- | --- | --- | --- | --- | --- | --- |
|  | N/ n (%) | HR (95% CI) |  | HR (95% CI) |  | HR (95% CI) |  | HR (95% CI) |  |
| **Demographics** | | | | | | | | |  |
| Age category (<18 y.o. (ref)) |  |  |  |  |  |  |  |  |  |
| 18 - 34 y.o. | 1 809/ 165 (9) | 1.04 (0.72; 1.48) | 0.85 | 0.99 (0.69; 1.43) | 1.00 | 1.06 (0.74; 1.53) | 0.733 | 1.08 (0.75; 1.54) | 0.696 |
| 35 - 50 y.o | 14 040/ 1 899 (14) | 1.57 (1.14; 2.19) | 0.007 | 1.51 (1.09; 2.11) | 0.014 | 1.85 (1.33; 2.57) | <0.001 | 1.86 (1.34; 2.59) | <0.001 |
| 51 - 70 y.o. | 75 434/ 19 076 (25) | 3.19 (2.31; 4.43) | <0.001 | 2.91 (2.1; 4.04) | <0.001 | 3.69 (2.66; 5.12) | <0.001 | 3.71 (2.67; 5.14) | <0.001 |
| > 70 y.o. | 46 833/ 23 233 (50) | 7.61 (5.49; 10.5) | <0.001 | 6.66 (4.8; 9.24) | <0.001 | 8.12 (5.86; 11.27) | <0.001 | 8.15 (5.87; 11.3) | <0.001 |
| Gender (Male vs Female (ref)) | 72 584/ 22 431 (31) | 0.91 (0.89; 0.92) | <0.001 | 1.12 (1.1; 1.14) | <0.001 | 1.12 (1.1; 1.14) | <0.001 | 1.12 (1.1; 1.14) | <0.001 |
| Ethnicity (Kazakh (ref)) |  |  |  |  |  |  |  |  |  |
| Russian | 41 978/ 17 481 (42) | 1.85 (1.82; 1.89) | <0.001 | 1.55 (1.52; 1.58) | <0.001 | 1.54 (1.51; 1.57) | <0.001 | 1.53 (1.49; 1.57) | <0.001 |
| Other | 26 347/ 8 969 (34) | 1.42 (1.38; 1.46) | <0.001 | 1.22 (1.19; 1.25) | <0.001 | 1.18 (1.15; 1.21) | <0.001 | 1.18 (1.15; 1.21) | <0.001 |
| Living area (Rural vs Urban (ref)) | 48 436/ 14 587 (30) | 0.88 (0.87; 0.9) | <0.001 | 1.06 (1.04; 1.08) | <0.001 | 1.05 (1.03; 1.07) | <0.001 | 1.05 (1.03; 1.07) | <0.001 |
| Admission (Urgent vs Elective (ref)) | 132 233/ 42 894 (32) |  | <0.001 | 1.34 (1.27; 1.41) | <0.001 | 1.34 (1.28; 1.41) | <0.001 | 1.35 (1.28; 1.42) | <0.001 |
| **Comorbidities** | | | | | | | | |  |
| Diabetes | 20 956/ 7 284 (35) | 1.12 (1.09; 1.15) | <0.001 |  |  | 1.34 (1.3; 1.37) | <0.001 | 1.34 (1.3; 1.37) | <0.001 |
| Hypertension | 70 477/ 17 893 (25) | 0.59 (0.58; 0.6) | <0.001 |  |  | 0.57 (0.56; 0.58) | <0.001 | 0.58 (0.57; 0.59) | <0.001 |
| **Surgery types** | | | | | | | | |  |
| Endovascular | 3 319/ 617 (19) | 0.56 (0.51; 0.6) | <0.001 |  |  |  |  | 0.63 (0.58; 0.69) | <0.001 |
| Trepanation and decompression | 98/ 53 (54) | 2.13 (1.63; 2.79) | <0.001 |  |  |  |  | 2.3 (1.75; 3.01) | <0.001 |
| Neuronavigational operation | 16/ 5 (31) | 0.76 (0.32; 1.82) | 0.537 |  |  |  |  | 0.77 (0.32; 1.85) | 0.56 |
| Shunt and anastomosis | 27/ 16 (59) | 2.24 (1.37; 3.66) | 0.001 |  |  |  |  | 1.82 (1.11; 2.98) | 0.017 |
| Cardiovascular | 538/ 400 (74) | 3.76 (3.41; 4.15) | <0.001 |  |  |  |  | 3.17 (2.88; 3.51) | <0.001 |

Model 1= adjusted to demographics (age, gender, ethnicity, admission, residency); Model 2 = Model 1 + comorbidities; Model 3 = Model 2 + surgery types.

##### Supplementary table 3. Association between socio-demographics and medical parameters and mortality of intracerebral hemorrhage (n=34,262) in the years of 2014-2019

a) in-hospital case-fatality rates

| Variable | Frequency/dead | Unadjusted | p-value | Model 1 | p-value | Model 2 | p-value | Model 3 | p-value | |  |
| --- | --- | --- | --- | --- | --- | --- | --- | --- | --- | --- | --- |
|  | N/ n (%) | HR (95% CI) |  | HR (95% CI) |  | HR (95% CI) |  | HR (95% CI) |  |  |  |
| **Demographics** | | | | | | | | | |  | |
| Age category (<18 y.o. (ref)) |  |  |  |  |  |  |  |  |  | |  |
| 18 - 34 y.o. | 809/ 168 (23) | 1.24 (0.99; 1.54) | 0.056 | 1.08 (0.87; 1.35) | 0.482 | 1.18 (0.95; 1.47) | 0.142 | 1.21 (0.97; 1.51) | 0.087 | |  |
| 35 - 50 y.o | 5 645/ 1 499 (26.6) | 1.65 (1.39; 1.94) | <0.001 | 1.43 (1.21; 1.69) | <0.001 | 1.85 (1.57; 2.19) | <0.001 | 1.88 (1.59; 2.22) | <0.001 | |  |
| 51 - 70 y.o. | 19 350/ 4 891 (25.3) | 1.55 (1.32; 1.82) | <0.001 | 1.32 (1.12; 1.55) | 0.001 | 1.79 (1.53; 2.11) | <0.001 | 1.79 (1.53; 2.12) | <0.001 | |  |
| > 70 y.o. | 7 560/ 2 208 (29.2) | 1.85 (1.57; 2.18) | <0.001 | 1.48 (1.26; 1.75) | <0.001 | 1.85 (1.57; 2.18) | <0.001 | 1.83 (1.55; 2.17) | <0.001 | |  |
| Gender (Male vs Female (ref)) | 18 603/ 4 889 (26.3) | 1.03 (0.98; 1.07) | 0.241 | 1.07 (1.02; 1.12) | 0.002 | 1.04 (0.99; 1.08) | 0.084 | 1.04 (0.99; 1.08) | 0.074 | |  |
| Ethnicity (Kazakh (ref)) |  |  |  |  |  |  |  |  |  | |  |
| Russian | 7 191/ 2 527 (35) | 1.66 (1.58; 1.74) | <0.001 | 1.59 (1.51; 1.67) | <0.001 | 1.51 (1.43; 1.58) | <0.001 | 1.50 (1.43; 1.58) | <0.001 | |  |
| Other | 5 403/ 1 461 (27) | 1.22 (1.15; 1.29) | <0.001 | 1.19 (1.12; 1.26) | <0.001 | 1.10 (1.04; 1.17) | 0.002 | 1.09 (1.03; 1.16) | 0.004 | |  |
| Living area (Rural vs Urban (ref)) | 14 483/ 3 529 (24.4) | 0.88 (0.85; 0.92) | <0.001 | 0.98 (0.94; 1.02) | 0.296 | 0.97 (0.93; 1.01) | 0.158 | 0.96 (0.92; 1.01) | 0.081 | |  |
| Admission (Urgent vs Elective (ref)) | 32 911/ 8 835 (26.8) | 4.89 (3.95; 6.05) | <0.001 | 4.51 (3.65; 5.59) | <0.001 | 4.53 (3.65; 5.61) | <0.001 | 4.59 (3.71; 5.69) | <0.001 | |  |
| **Comorbidities** | | | | | | | | | |  | |
| Diabetes | 2 217/ 520 (23.5) | 0.88 (0.80; 0.96) | 0.004 |  |  | 1.13 (1.03; 1.24) | 0.008 | 1.13 (1.03; 1.24) | 0.008 | |  |
| Hypertension | 13 919/ 2 307 (16.6) | 0.46 (0.44; 0.49) | <0.001 |  |  | 0.45 (0.43; 0.48) | <0.001 | 0.46 (0.44; 0.48) | <0.001 | |  |
| **Surgery types** | | | | | | | | | |  | |
| Endovascular | 810/ 101 (12.5) | 0.42 (0.34; 0.51) | <0.001 |  |  |  |  | 0.44 (0.36; 0.54) | <0.001 | |  |
| Trepanation and decompression | 2 259/ 595 (26.3) | 0.95 (0.87; 1.03) | 0.206 |  |  |  |  | 0.89 (0.81; 0.96) | 0.005 | |  |
| Neuronavigational operation | 286/ 44 (15.4) | 0.52 (0.39; 0.70) | <0.001 |  |  |  |  | 0.49 (0.37; 0.67) | <0.001 | |  |
| Shunt and anastomosis | 147/ 47 (32) | 1.06 (0.79; 1.41) | 0.682 |  |  |  |  | 1.15 (0.86; 1.53) | 0.343 | |  |
| Cardiovascular | 385/ 170 (44.2) | 1.41 (1.21; 1.64) | <0.001 |  |  |  |  | 1.24 (1.06; 1.44) | 0.006 | |  |

Model 1= adjusted to demographics (age, gender, ethnicity, admission, residency); Model 2 = Model 1 + comorbidities; Model 3 = Model 2 + surgery types.

b) 30-day all-cause mortality rates

| Variable | Frequency/dead | Unadjusted | p-value | Model 1 | p-value | Model 2 | p-value | Model 3 | p-value |
| --- | --- | --- | --- | --- | --- | --- | --- | --- | --- |
|  | N/ n (%) | HR (95% CI) |  | HR (95% CI) |  | HR (95% CI) |  | HR (95% CI) |  |
| **Demographics** | | | | | | | | |  |
| Age category (<18 y.o. (ref)) |  |  |  |  |  |  |  |  |  |
| 18 - 34 y.o. | 809/ 169 (20.9) | 1.34 (1.07; 1.67) | 0.011 | 1.18 (0.94; 1.47) | 0.151 | 1.29 (1.03; 1.61) | 0.026 | 1.32 (1.06; 1.65) | 0.014 |
| 35 - 50 y.o | 5 645/ 1 515 (26.8) | 1.78 (1.50; 2.12) | <0.001 | 1.55 (1.31; 1.84) | <0.001 | 2.04 (1.71; 2.42) | <0.001 | 2.06 (1.74; 2.45) | <0.001 |
| 51 - 70 y.o. | 19 350/ 5 131 (26.5) | 1.74 (1.47; 2.05) | <0.001 | 1.47 (1.25; 1.74) | <0.001 | 2.04 (1.73; 2.41) | <0.001 | 2.04 (1.73; 2.42) | <0.001 |
| > 70 y.o. | 7 560/ 2 801 (37.1) | 2.51 (2.12; 2.96) | <0.001 | 2.00 (1.69; 2.37) | <0.001 | 2.52 (2.13; 2.99) | <0.001 | 2.49 (2.11; 2.96) | <0.001 |
| Gender (Male vs Female (ref)) | 18 603/ 5 201 (28) | 0.96 (0.93; 1.00) | 0.076 | 1.04 (0.99; 1.08) | 0.087 | 1.01 (0.97; 1.05) | 0.794 | 1.01 (0.97; 1.05) | 0.705 |
| Ethnicity (Kazakh (ref)) |  |  |  |  |  |  |  |  |  |
| Russian | 7 191/ 2 749 (38.2) | 1.65 (1.58; 1.73) | <0.001 | 1.56 (1.49; 1.64) | <0.001 | 1.48 (1.41; 1.55) | <0.001 | 1.48 (1.41; 1.55) | <0.001 |
| Other | 5 403/ 1 616 (30) | 1.23 (1.17; 1.30) | <0.001 | 1.19 (1.12; 1.25) | <0.001 | 1.09 (1.04; 1.16) | 0.001 | 1.09 (1.03; 1.15) | 0.002 |
| Living area (Rural vs Urban (ref)) | 14 483/ 4 042 (27.9) | 0.95 (0.92; 0.99) | 0.023 | 1.06 (1.02; 1.11) | 0.004 | 1.05 (1.01; 1.09) | 0.015 | 1.04 (1.00; 1.09) | 0.044 |
| Admission (Urgent vs Elective (ref)) | 32 911/ 9 700 (29.5) | 7.59 (5.86; 9.78) | <0.001 | 6.85 (5.31; 8.83) | <0.001 | 6.87 (5.33; 8.85) | <0.001 | 6.99 (5.42; 9.01) | <0.001 |
| **Comorbidities** | | | | | | | | |  |
| Diabetes | 2 217/ 566 (25.5) | 0.87 (0.80; 0.95) | 0.002 |  |  | 1.14 (1.04; 1.24) | 0.004 | 1.14 (1.04; 1.24) | 0.004 |
| Hypertension | 13 919/ 2 434 (17.5) | 0.44 (0.42; 0.46) | <0.001 |  |  | 0.43 (0.41; 0.45) | <0.001 | 0.43 (0.41; 0.46) | <0.001 |
| **Surgery types** | | | | | | | | |  |
| Endovascular | 810/ 107 (13.2) | 0.40 (0.33; 0.49) | <0.001 |  |  |  |  | 0.44 (0.36; 0.53) | <0.001 |
| Trepanation and decompression | 2 259/ 615 (27.2) | 0.89 (0.82; 0.96) | 0.005 |  |  |  |  | 0.87 (0.79; 0.94) | 0.001 |
| Neuronavigational operation | 286/ 47 (16.4) | 0.51 (0.38; 0.68) | <0.001 |  |  |  |  | 0.49 (0.37; 0.66) | <0.001 |
| Shunt and anastomosis | 147/ 47 (32) | 0.96 (0.72; 1.28) | 0.801 |  |  |  |  | 1.11 (0.83; 1.49) | 0.467 |
| Cardiovascular | 385/ 168 (43.6) | 1.26 (1.09; 1.47) | 0.003 |  |  |  |  | 1.13 (0.97; 1.32) | 0.119 |

Model 1= adjusted to demographics (age, gender, ethnicity, admission, residency); Model 2 = Model 1 + comorbidities; Model 3 = Model 2 + surgery types.

c) all-cause mortality rates

| Variable | Frequency/dead | Unadjusted | p-value | Model 1 | p-value | Model 2 | p-value | Model 3 | p-value |
| --- | --- | --- | --- | --- | --- | --- | --- | --- | --- |
|  | N/ n (%) | HR (95% CI) |  | HR (95% CI) |  | HR (95% CI) |  | HR (95% CI) |  |
| **Demographics** | | | | | | |  |  |  |
| Age category (<18 y.o. (ref)) |  |  |  |  |  |  |  |  |  |
| 18 - 34 y.o. | 809/ 200 (25) | 1.25 (1.03; 1.53) | 0.027 | 1.14 (0.93; 1.39) | 0.212 | 1.22 (0.99; 1.48) | 0.056 | 1.24 (1.02; 1.52) | 0.034 |
| 35 - 50 y.o | 5 645/ 1 88 (33) | 1.81 (1.56; 2.11) | <0.001 | 1.63 (1.4; 1.9) | <0.001 | 2.01 (1.73; 2.35) | <0.001 | 2.04 (1.75; 2.37) | <0.001 |
| 51 - 70 y.o. | 19 350/ 7 286 (38) | 2.06 (1.78; 2.38) | <0.001 | 1.82 (1.57; 2.11) | <0.001 | 2.32 (2.0; 2.69) | <0.001 | 2.32 (1.99; 2.69) | <0.001 |
| > 70 y.o. | 7 560/ 4 261 (56) | 3.39 (2.92; 3.93) | <0.001 | 2.86 (2.47; 3.32) | <0.001 | 3.41 (2.93; 3.95) | <0.001 | 3.37 (2.9; 3.92) | <0.001 |
| Gender (Male vs Female (ref)) | 18 603/ 7 352 (40) | 0.95 (0.92; 0.99) | 0.007 | 1.05 (1.02; 1.09) | 0.003 | 1.03 (0.99; 1.07) | 0.067 | 1.03 (0.99; 1.07) | 0.052 |
| Ethnicity (Kazakh (ref)) |  |  |  |  |  |  |  |  |  |
| Russian | 7 191/ 3 728 (52) | 1.61 (1.55; 1.67) | <0.001 | 1.47 (1.41; 1.53) | <0.001 | 1.42 (1.37; 1.48) | <0.001 | 1.42 (1.36; 1.48) | <0.001 |
| Other | 5 403/ 2 236 (41) | 1.19 (1.13; 1.24) | <0.001 | 1.12 (1.06; 1.17) | <0.001 | 1.05 (1.004; 1.1) | 0.033 | 1.05 (0.99; 1.1) | 0.059 |
| Living area (Rural vs Urban (ref)) | 14 483/ 5 688 (39) | 0.94 (0.91; 0.97) | <0.001 | 1.04 (1.0001; 1.07) | 0.045 | 1.03 (0.99; 1.07) | 0.088 | 1.02 (0.99; 1.06) | 0.191 |
| Admission (Urgent vs Elective (ref)) | 32 911/ 13 549 (41) | 2.64 (2.34; 2.97) | <0.001 | 2.32 (2.05; 2.62) | <0.001 | 2.33 (2.07; 2.63) | <0.001 | 2.36 (2.1; 2.67) | <0.001 |
| **Comorbidities** | | | | | | |  |  |  |
| Diabetes | 2 217/ 948 (43) | 1.05 (0.98; 1.12) | 0.167 |  |  | 1.26 (1.18; 1.35) | <0.001 | 1.26 (1.18; 1.35) | <0.001 |
| Hypertension | 13 919/ 4 237 (30) | 0.57 (0.55; 0.59) | <0.001 |  |  | 0.55 (0.53; 0.57) | <0.001 | 0.55 (0.53; 0.57) | <0.001 |
| **Surgery types** | | | | | | |  |  |  |
| Endovascular | 810/ 187 (23) | 0.49 (0.43; 0.58) | <0.001 |  |  |  |  | 0.55 (0.48; 0.64) | <0.001 |
| Trepanation and decompression | 2 259/ 838 (37) | 0.87 (0.81; 0.93) | <0.001 |  |  |  |  | 0.87 (0.81; 0.94) | <0.001 |
| Neuronavigational operation | 286/ 66 (23) | 0.49 (0.39; 0.63) | <0.001 |  |  |  |  | 0.49 (0.39; 0.64) | <0.001 |
| Shunt and anastomosis | 147/ 62 (42) | 0.94 (0.73; 1.21) | 0.637 |  |  |  |  | 1.16 (0.9; 1.49) | 0.244 |
| Cardiovascular | 385/ 222 (58) | 1.27 (1.11; 1.45) | <0.001 |  |  |  |  | 1.19 (1.04; 1.36) | 0.01 |

Model 1= adjusted to demographics (age, gender, ethnicity, admission, residency); Model 2 = Model 1 + comorbidities; Model 3 = Model 2 + surgery types.

#####

##### Supplementary table 4. Association between socio-demographics and medical parameters and mortality of subarachnoid hemorrhage (n=5,143) in the years of 2014-2019

a) in-hospital case-fatality rates

| Variable | Frequency/dead | Unadjusted | p-value | Model 1 | p-value | Model 2 | p-value | Model 3 | p-value |
| --- | --- | --- | --- | --- | --- | --- | --- | --- | --- |
|  | N/ n (%) | HR (95% CI) |  | HR (95% CI) |  | HR (95% CI) |  | HR (95% CI) |  |
| **Demographics** | | | | | | | | |  |
| Age category (<18 y.o. (ref)) |  |  |  |  |  |  |  |  |  |
| 18 - 34 y.o. | 329/ 61 (18.5) | 1.71 (1.16; 2.52) | 0.006 | 1.53 (1.04; 2.26) | 0.03 | 1.61 (1.09; 2.37) | 0.016 | 1.75 (1.19; 2.58) | 0.005 |
| 35 - 50 y.o | 1 173/ 259 (22.1) | 2.07 (1.51; 2.84) | <0.001 | 1.82 (1.32; 2.50) | <0.001 | 2.31 (1.68; 3.19) | <0.001 | 2.55 (1.85; 3.52) | <0.001 |
| 51 - 70 y.o. | 2 512/ 650 (25.9) | 2.51 (1.86; 3.39) | <0.001 | 2.24 (1.65; 3.04) | <0.001 | 3.12 (2.29; 4.24) | <0.001 | 3.36 (2.47; 4.57) | <0.001 |
| > 70 y.o. | 726/ 234 (32.2) | 3.37 (2.45; 4.64) | <0.001 | 2.95 (2.13; 4.08) | <0.001 | 3.69 (2.66; 5.10) | <0.001 | 3.79 (2.74; 5.26) | <0.001 |
| Gender (Male vs Female (ref)) | 2 505/ 632 (25.2) | 1.09 (0.98; 1.22) | 0.112 | 1.20 (1.08; 1.35) | 0.001 | 1.12 (1.01; 1.26) | 0.047 | 1.10 (0.98; 1.23) | 0.091 |
| Ethnicity (Kazakh (ref)) |  |  |  |  |  |  |  |  |  |
| Russian | 1 020/ 315 (30.9) | 1.50 (1.32; 1.72) | <0.001 | 1.37 (1.19; 1.57) | <0.001 | 1.36 (1.19; 1.56) | <0.001 | 1.34 (1.17; 1.54) | <0.001 |
| Other | 810/ 218 (26.9) | 1.29 (1.11; 1.51) | 0.001 | 1.19 (1.02; 1.38) | 0.028 | 1.13 (0.97; 1.32) | 0.117 | 1.11 (0.95; 1.29) | 0.174 |
| Living area (Rural vs Urban (ref)) | 2 384/ 582 (24.4) | 1.02 (0.91; 1.14) | 0.731 | 1.08 (0.96; 1.21) | 0.184 | 1.08 (0.96; 1.21) | 0.209 | 1.05 (0.94; 1.18) | 0.406 |
| Admission (Urgent vs Elective (ref)) | 4 818/ 1 236 (25.7) | 7.36 (4.26; 12.71) | <0.001 | 6.71 (3.88; 11.6) | <0.001 | 6.43 (3.72; 11.1) | <0.001 | 6.41 (3.71; 11.1) | <0.001 |
| **Comorbidities** | | | | | | | | |  |
| Diabetes | 245/ 48 (19.6) | 0.79 (0.59; 1.05) | 0.106 |  |  | 0.97 (0.73; 1.31) | 0.858 | 0.96 (0.72; 1.29) | 0.794 |
| Hypertension | 1 822/ 270 (14.8) | 0.46 (0.40; 0.53) | <0.001 |  |  | 0.41 (0.35; 0.47) | <0.001 | 0.42 (0.36; 0.48) | <0.001 |
| **Surgery types** | | | | | | | | |  |
| Endovascular | 486/ 52 (10.7) | 0.38 (0.29; 0.49) | <0.001 |  |  |  |  | 0.39 (0.29; 0.52) | <0.001 |
| Trepanation and decompression | 64/ 27 (42.2) | 1.57 (1.07; 2.29) | 0.021 |  |  |  |  | 1.47 (0.99; 2.15) | 0.051 |
| Neuronavigational operation | 8/3 (37.5) | 1.75 (0.56; 5.44) | 0.333 |  |  |  |  | 2.03 (0.65; 6.32) | 0.223 |
| Shunt and anastomosis | 14/ 7 (50) | 1.83 (0.87; 3.84) | 0.112 |  |  |  |  | 2.24 (1.06; 4.74) | 0.035 |
| Cardiovascular | 30/ 15 (50) | 1.77 (1.07; 2.95) | 0.027 |  |  |  |  | 1.43 (0.86; 2.39) | 0.167 |

Model 1= adjusted to demographics (age, gender, ethnicity, admission, residency); Model 2 = Model 1 + comorbidities; Model 3 = Model 2 + surgery types.

b) 30-day all-cause mortality rates

| Variable | Frequency/dead | Unadjusted | p-value | Model 1 | p-value | Model 2 | p-value | Model 3 | p-value |
| --- | --- | --- | --- | --- | --- | --- | --- | --- | --- |
|  | N/ n (%) | HR (95% CI) |  | HR (95% CI) |  | HR (95% CI) |  | HR (95% CI) |  |
| **Demographics** | | | | | | | | |  |
| Age category (<18 y.o. (ref)) |  |  |  |  |  |  |  |  |  |
| 18 - 34 y.o. | 329/ 60 (18.2) | 1.64 (1.12; 2.41) | 0.011 | 1.48 (1.01; 2.18) | 0.046 | 1.55 (1.06; 2.29) | 0.025 | 1.68 (1.14; 2.47) | 0.009 |
| 35 - 50 y.o | 1 173/ 262 (22.3) | 2.04 (1.49; 2.79) | <0.001 | 1.79 (1.31; 2.46) | <0.001 | 2.29 (1.68; 3.15) | <0.001 | 2.51 (1.83; 3.45) | <0.001 |
| 51 - 70 y.o. | 2 512/ 700 (27.9) | 2.63 (1.95; 3.55) | <0.001 | 2.34 (1.74; 3.17) | <0.001 | 3.29 (2.44; 4.46) | <0.001 | 3.52 (2.60; 4.77) | <0.001 |
| > 70 y.o. | 726/ 301 (41.5) | 4.22 (3.09; 5.75) | <0.001 | 3.67 (2.68; 5.03) | <0.001 | 4.62 (3.37; 6.33) | <0.001 | 4.74 (3.45; 6.50) | <0.001 |
| Gender (Male vs Female (ref)) | 2 505/ 665 (26.5) | 1.01 (0.91; 1.12) | 0.838 | 1.14 (1.02; 1.27) | 0.018 | 1.06 (0.95; 1.18) | 0.293 | 1.04 (0.94; 1.16) | 0.449 |
| Ethnicity (Kazakh (ref)) |  |  |  |  |  |  |  |  |  |
| Russian | 1 020/ 334 (32.7) | 1.44 (1.26; 1.63) | <0.001 | 1.31 (1.15; 1.49) | <0.001 | 1.29 (1.13; 1.48) | <0.001 | 1.27 (1.12; 1.45) | <0.001 |
| Other | 810/ 241 (29.8) | 1.29 (1.11; 1.49) | 0.001 | 1.17 (1.01; 1.35) | 0.035 | 1.11 (0.96; 1.29) | 0.158 | 1.09 (0.95; 1.27) | 0.211 |
| Living area (Rural vs Urban (ref)) | 2 384/ 655 (27.5) | 1.07 (0.97; 1.19) | 0.192 | 1.13 (1.01; 1.26) | 0.03 | 1.12 (1.01; 1.25) | 0.038 | 1.09 (0.98; 1.23) | 0.096 |
| Admission (Urgent vs Elective (ref)) | 4 818/ 1 357 (28.2) | 8.72 (4.94; 15.4) | <0.001 | 7.89 (4.47; 13.9) | <0.001 | 7.56 (4.28; 13.4) | <0.001 | 7.54 (4.27; 13.3) | <0.001 |
| **Comorbidities** | | | | | | | | |  |
| Diabetes | 245/ 53 (21.6) | 0.79 (0.60; 1.05) | 0.1 |  |  | 0.97 (0.73; 1.28) | 0.811 | 0.96 (0.72; 1.26) | 0.748 |
| Hypertension | 1 822/ 291 (16) | 0.45 (0.39; 0.51) | <0.001 |  |  | 0.39 (0.34; 0.45) | <0.001 | 0.40 (0.35; 0.46) | <0.001 |
| **Surgery types** | | | | | | | | |  |
| Endovascular | 486/ 61 (12.6) | 0.40 (0.31; 0.52) | <0.001 |  |  |  |  | 0.44 (0.34; 0.57) | <0.001 |
| Trepanation and decompression | 64/ 28 (43.8) | 1.48 (1.02; 2.15) | 0.039 |  |  |  |  | 1.42 (0.98; 2.08) | 0.066 |
| Neuronavigational operation | 8/3 (37.5) | 1.61 (0.52; 5.00) | 0.409 |  |  |  |  | 2.00 (0.64; 6.24) | 0.231 |
| Shunt and anastomosis | 14/ 7 (50) | 1.67 (0.79; 3.50) | 0.177 |  |  |  |  | 1.93 (0.92; 4.08) | 0.084 |
| Cardiovascular | 30/ 16 (53.3) | 1.71 (1.04; 2.79) | 0.033 |  |  |  |  | 1.42 (0.86; 2.32) | 0.168 |

Model 1= adjusted to demographics (age, gender, ethnicity, admission, residency); Model 2 = Model 1 + comorbidities; Model 3 = Model 2 + surgery types.

c) all-cause mortality rates

| Variable | Frequency/dead | Unadjusted | p-value | Model 1 | p-value | Model 2 | p-value | Model 3 | p-value |
| --- | --- | --- | --- | --- | --- | --- | --- | --- | --- |
|  | N/ n (%) | HR (95% CI) |  | HR (95% CI) |  | HR (95% CI) |  | HR (95% CI) |  |
| **Demographics** | | | | | | | | |  |
| Age category (<18 y.o. (ref)) |  |  |  |  |  |  |  |  |  |
| 18 - 34 y.o. | 329/ 73 (22) | 1.69 (1.19; 2.38) | 0.003 | 1.56 (1.1; 2.21) | 0.012 | 1.63 (1.15; 2.31) | 0.006 | 1.76 (1.24; 2.49) | 0.001 |
| 35 - 50 y.o | 1 173/ 336 (29) | 2.25 (1.69; 2.97) | <0.001 | 2.05 (1.55; 2.72) | <0.001 | 2.58 (1.94; 3.43) | <0.001 | 2.81 (2.11; 3.73) | <0.001 |
| 51 - 70 y.o. | 2 512/ 928 (37) | 3.04 (2.32; 3.98) | <0.001 | 2.81 (2.15; 3.68) | <0.001 | 3.82 (2.92: 5.02) | <0.001 | 4.07 (3.1; 5.35) | <0.001 |
| > 70 y.o. | 726/ 439 (60) | 5.71 (4.33; 7.53) | <0.001 | 5.22 (3.95; 6.91) | <0.001 | 6.46 (4.87; 8.55) | <0.001 | 6.6 (4.98; 8.75) | <0.001 |
| Gender (Male vs Female (ref)) | 2 505/ 909 (36) | 1.04 (0.95; 1.14) | 0.387 | 1.19 (1.09; 1.31) | <0.001 | 1.12 (1.02; 1.23) | 0.015 | 1.1 (1.005; 1.21) | 0.038 |
| Ethnicity (Kazakh (ref)) |  |  |  |  |  |  |  |  |  |
| Russian | 1 020/ 446 (44) | 1.46 (1.31; 1.63) | <0.001 | 1.32 (1.18; 1.48) | <0.001 | 1.32 (1.17; 1.48) | <0.001 | 1.29 (1.15; 1.45) | <0.001 |
| Other | 810/ 315 (39) | 1.27 (1.12; 1.44) | <0.001 | 1.14 (1.004; 1.29) | 0.043 | 1.09 (0.96; 1.23) | 0.206 | 1.07 (0.94; 1.21) | 0.303 |
| Living area (Rural vs Urban (ref)) | 2 384/ 888 (37) | 1.09 (1.003; 1.2) | 0.044 | 1.15 (1.04; 1.26) | 0.005 | 1.14 (1.04; 1.26) | 0.005 | 1.11 (1.01; 1.23) | 0.025 |
| Admission (Urgent vs Elective (ref)) | 4 818/ 1 780 (37) | 2.84 (2.16; 3.73) | <0.001 | 2.55 (1.93; 3.35)\ | <0.001 |  | <0.001 | 2.48 (1.88; 3.26) | <0.001 |
| **Comorbidities** | | | | | | | | |  |
| Diabetes | 245/ 82 (33) | 0.91 (0.72; 1.14) | 0.406 |  |  | 1.03 (0.82; 1.29) | 0.815 | 1.02 (0.81; 1.27) | 0.897 |
| Hypertension | 1 822/ 456 (25) | 0.53 (0.48; 0.59) | <0.001 |  |  | 0.46 (0.41; 0.51) | <0.001 | 0.47 (0.42; 0.52) | <0.001 |
| **Surgery types** | | | | | | | | |  |
| Endovascular | 486/ 77 (16) | 0.38 (0.3; 0.48) | <0.001 |  |  |  |  | 0.42 (0.33; 0.53) | <0.001 |
| Trepanation and decompression | 64/ 35 (55) | 1.45 (1.04; 2.03) | 0.028 |  |  |  |  | 1.48 (1.05; 2.07) | 0.023 |
| Neuronavigational operation | 8/3 (38) | 1.2 (0.39; 3.73) | 0.749 |  |  |  |  | 1.68 (0.54; 5.24) | 0.369 |
| Shunt and anastomosis | 14/ 7 (50) | 1.3 (0.62; 2.74) | 0.485 |  |  |  |  | 1.55 (0.73; 3.26) | 0.252 |
| Cardiovascular | 30/ 20 (67) | 1.77 (1.14; 2.74) | 0.011 |  |  |  |  | 1.5 (0.96; 2.34) | 0.073 |

Model 1= adjusted to demographics (age, gender, ethnicity, admission, residency); Model 2 = Model 1 + comorbidities; Model 3 = Model 2 + surgery types.

#####

Supplementary figure 5. Kaplan-Meier survival curves due to in-hospital case-fatality based on discharge status

a) 30-day in-hospital case-fatality by stroke type


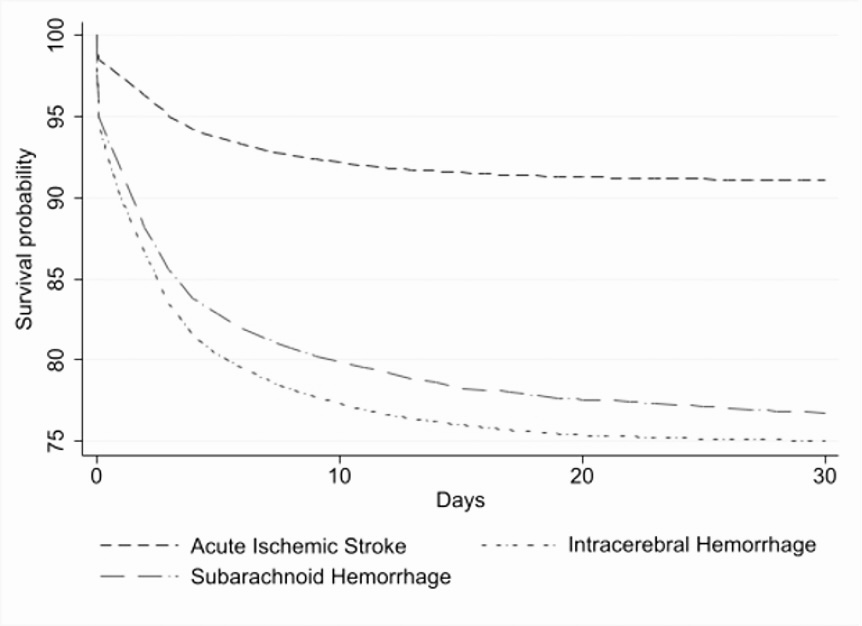


b) 30-day in-hospital case-fatality by diagnosis type adjusted for age and gender

#####
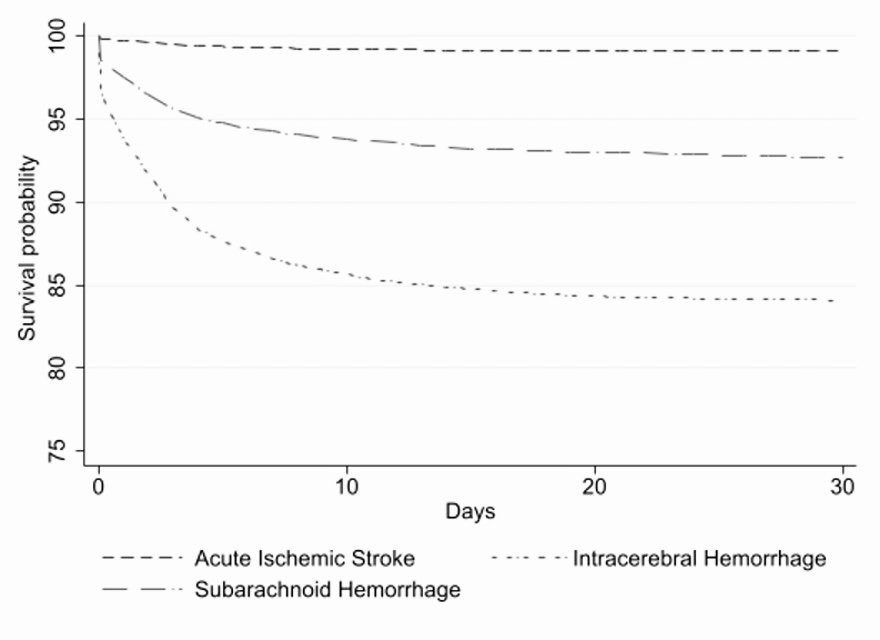

Supplement: Supplementary file 1 — Supplementary Information. [file 41598_2022_20302_MOESM1_ESM.docx]
